# Within-cycle instantaneous frequency profiles report oscillatory waveform dynamics

**DOI:** 10.1152/jn.00201.2021

**Published:** 2021-08-18

**Authors:** Andrew J. Quinn, Vítor Lopes-dos-Santos, Norden Huang, Wei-Kuang Liang, Chi-Hung Juan, Jia-Rong Yeh, Anna C. Nobre, David Dupret, Mark W. Woolrich

**Affiliations:** 1 Oxford Centre for Human Brain Activity, Wellcome Centre for Integrative Neuroimaging, Department of Psychiatry, University of Oxford, OX3 7JX. UK; 2 Medical Research Council Brain Network Dynamics Unit, Nuffield Department of Clinical Neurosciences, University of Oxford, Oxford, OX1 3TH, UK; 3 Data Analysis and Application Laboratory, Innovation Centre, The First Institute of Oceanography, Qingdao, China; 4 Pilot National Laboratory for Marine Science and Technology, Qingdao, China; 5 Cognitive Intelligence and Precision Healthcare Centre, National Central University, Taiwan; 6 Institute of Cognitive Neuroscience, National Central University, Taoyuan City, Taiwan; 7 Department of Experimental Psychology, University of Oxford, Oxford. OX2 6GG. UK

**Keywords:** Waveform Shape, Oscillations, Non-sinusoidal, EMD, Instantaneous Frequency

## Abstract

Non-sinusoidal waveform is emerging as an important feature of neuronal oscillations. However, the role of single cycle shape dynamics in rapidly unfolding brain activity remains unclear. Here, we develop an analytical framework that isolates oscillatory signals from time-series using masked Empirical Mode Decomposition to quantify dynamical changes in the shape of individual cycles (along with amplitude, frequency and phase) using instantaneous frequency. We show how phase-alignment, a process of projecting cycles into a regularly sampled phase-grid space, makes it possible to compare cycles of different durations and shapes. ‘Normalised shapes’ can then be constructed with high temporal detail whilst accounting for differences in both duration and amplitude. We find that the instantaneous frequency tracks non-sinusoidal shapes in both simulated and real data. Notably, in local field potential recordings of mouse hippocampal CA1, we find that theta oscillations have a stereotyped slow-descending slope in the cycle-wise average, yet exhibiting high variability on a cycle-by-cycle basis. We show how Principal Components Analysis allows identification of motifs of theta cycle waveform that have distinct associations to cycle amplitude, cycle duration and animal movement speed. By allowing investigation into oscillation shape at high temporal resolution, this analytical framework will open new lines of enquiry into how neuronal oscillations support moment-by-moment information processing and integration in brain networks.

## 1 Introduction

Frequency, phase and amplitude have long been reported as important features of neuronal oscillations with behavioural and electrophysiological relevance. Furthermore, neuronal oscillations show non-sinusoidal waveform shapes that span a wide range of spatial and temporal scales (Cole and Voytek, 2017). Whilst waveform shape is emerging as a fourth relevant feature of neuronal oscillations, many theories of neuronal oscillations currently assume sinusoidal waveforms. This might be due to the fact that characterising and quantifying non-sinusoidal waveforms remains a substantial analytic challenge (1, 2). To uncover the role of waveform dynamics in rapidly unfolding brain activity, there is a growing need for novel analysis methods that are able to characterise a wide range of waveform shape features at the single-cycle level.

Waveform shape related parameters, such as skewness or asymmetry, can be estimated from higher order Fourier spectra such as the bispectrum or bicoherence (3–5). These methods require relatively long data segments to have high frequency resolution, and therefore do not provide single-cycle estimates. Alternatively, a set of waveform features for individual cycles can be described by the relative durations of different quartiles of a cycle (6–8). Control point based analyses are clear, flexible and tractable on single cycles but have the drawback that each individual feature must be defined *a priori* and be based on a limited number of cycle control points such as the extrema and zero-crossings.

The temporal dynamics in oscillatory frequency can be quantified for a given waveform by its instantaneous frequency computed from the differential of the signal’s instantaneous phase (9, 10). Such instantaneous frequency estimates have been used previously in electrophysiology to explore dynamics in oscillatory peak frequency at high temporal resolution (11–14). Crucially, any non-sinusoidal waveform features in an oscillation will lead to within-cycle instantaneous frequency modulations in which the frequency of an oscillation changes from moment-to-moment within a single cycle (15). The degree of non-linearity of an oscillation is related to the total amount of within-cycle frequency modulation (16–19).

Accordingly, here we introduce a novel approach that creates waveform shape profiles to describe non-sinusoidal features in single cycles with high temporal details. To this end, we first operationalise waveform shape as the profile of instantaneous frequency across the cycle’s instantaneous phase. We then identify when and how an ongoing cycle deviates from a sinusoidal waveform by identifying points in the cycle where instantaneous frequency departs from a flat profile. Notably, a cycle with a wide peak has a relatively low instantaneous frequency around the peak, and a cycle with a fast-ascending edge has a relatively high instantaneous frequency between the trough and peak. Moreover, between cycle comparison requires to account for how different cycles of an oscillation play out at different speeds, leading to differences in extrema timing and overall duration. To overcome these problems, we thus also introduce the process of ‘phase-alignment’, which re-registers the instantaneous frequency profiles onto a regularly sampled set of points in the phase space.

To obtain the instantaneous phase time course of each cycle, we use the Empirical Mode Decomposition (EMD). EMD decomposes the time-series of interest into its oscillatory modes (Intrinsic Mode Functions; IMFs) that retain the non-stationary and non-linear signal features. The EMD and instantaneous frequency analysis are a promising tool for neuroscience and electrophysiology (12). The EMD has successfully been applied to electrophysiology data in a range of contexts including (but not limited to) rodent hippocampal theta oscillations (20), epileptic activity in human patients (21), TMS evoked EEG responses (22) and for assessing instantaneous phase-synchrony in intracranial EEG (23). We build on this prior work to introduce phase-aligned instantaneous frequency as a general measure of oscillatory waveform shape.

We outline and validate our novel approach in simulated data before applying it to theta-band oscillations recorded in the local field potentials (LFPs) of the mouse hippocampal CA1 during active exploratory behaviour. The hippocampal theta rhythm has a characteristic non-sinusoidal waveform shape (6, 24, 25) that is modulated by movement (5) and changes in sleep or drug states (24). Using EMD to identify the theta rhythm, we show that phase-aligned instantaneous frequency is able to robustly characterise a continuous waveform shape profile for hippocampal theta. The results confirm the stereotyped fast-ascending and slow-descending shape in the cycle-wise average. Yet, the shape of single cycles of theta appears to be highly variable. We describe this variability using a set of data-driven shape ‘motifs’. Finally, we find that cycle-level shape motifs have differential associations with theta amplitude, theta cycle duration and mouse movement speed. Overall, these findings show that behaviourally relevant dynamics in single-cycle oscillatory waveforms can be accurately and intuitively explored with phase-aligned instantaneous frequency profiles.

## 2 Materials and Methods

### 2.1 Data and Code Availability Statements

Code for the analyses in this paper are freely available online (https://github.com/OHBA-analysis/Quinn2021_Waveform) and data are available from the MRC BNDU Data Sharing Platform (https://data.mrc.ox.ac.uk/data-set/instantaneous-frequency-profiles-theta-cycles; requires free registration). The analyses in this study were carried out in python 3.7 using v0.4.0 of the EMD package (Quinn et al., 2021; https://emd.readthedocs.io/) and glmtools v0.1.0 for General Linear Model design and fitting (https://pypi.org/project/glmtools/). The wavelet transforms and principal components analysis were computed using SAILS v1.1.1 (27). The underlying python dependencies were numpy (28) and scipy (29) for computation and matplotlib (30) for visualisation.

### 2.2 Masked Empirical Mode Decomposition Methods

The EMD is implemented as a sifting algorithm that incrementally extracts the highest frequency features of a time-series into its oscillatory components known as IMFs (Huang et al., 1998). Once identified, the IMF is subtracted from the signal and the sifting process repeated to find the next fastest set of oscillatory dynamics. This process is iterated through until only a residual trend remains in the dataset, constituting the very slowest dynamics of the signal.

Transient or intermittent oscillatory signals can lead to a mix of different frequency components appearing in a single IMF; an issue known as mode mixing (31, 32). Specifically, the sift algorithm always look to identify the highest frequency component in a signal; however, if the high frequency oscillation is transient, then lower frequency oscillations may jump into the IMF during time periods when the high frequency signal is not present. To reduce this mode mixing, we use an adapted version of the mask sift (17, 31). The mEMD involves the same core process as the original sift outlined above. However, at each iteration, a masking signal is added to the data before all the extrema (maxima and minima) in the masked signal are identified. The mask is a simple sinusoid which acts to prevent lower frequency components from entering an IMF. During time periods with high frequency activity, the mask sift will return the sum of the high frequency signal and the mask. As the mask is known, it can be removed by simple subtraction. When there is no high frequency activity the mask sift will return only the mask signal. As such, the mask signal is able to prevent mixing between transient oscillatory components (Deering and Kaiser, 2005; Fosso and Molinas, 2017). The mask sift algorithm is described in pseudocode and a flowchart in supplemental section 8.1 (https://doi.org/10.6084/m9.figshare.15028986)

The performance of the sift is limited by a number of factors such as accuracy in peak detection and overshoot or edge effects in the envelope interpolation. As such, when applied to real data, the sift may not always perfectly isolate individual oscillations. Specifically, the EMD algorithm depends upon accurate estimation of the local mean of a signal which is limited by a number of factors in real data analysis. The most critical step is ensuring accurate upper and lower envelope estimation. The cubic spline is typically used for this though it does not preserve monotonicity between the original and interpolated signal. For example, if three points are strictly ascending in the original signal the cubic spline cannot guarantee that the interpolated signal will also be strictly ascending. This can lead to parts of the interpolation exaggerating dynamics in the envelope by causing overshoot and potentially leading to the upper and lower envelopes crossing over. The monotonic PCHIP (33) interpolation does preserve monotonicity and provides a more stable envelope is noisy data (See supplemental figure S2 for an illustration; https://doi.org/10.6084/m9.figshare.15028986).

#### 2.2.1 Frequency Transformation

The analytic form of each IMF was constructed using the Hilbert transform and the instantaneous phase set as the angle of the analytic form on the complex plane (See Figure 2C for an example). To attenuate noise in the phase estimation, the unwrapped phase time-course was smoothed using a Savitsky-Golay filter (scipy.signal.savgol_filter; order = 1, window size = 3 samples). The instantaneous frequency (See Figure 2D for an example) in Hertz is then computed from the derivative of this unwrapped phase:

$$\text{IF}=\frac{\text{fs}}{2\pi }\frac{\text{d}\emptyset (\text{t})}{\text{dt}}$$

Where fs is the sampling frequency, t is time in samples and Ø(t) is the unwrapped instantaneous phase time-course. The instantaneous amplitude time-course is computed as the absolute value of the analytic form of each IMF.

The distribution of instantaneous amplitude values by frequency or time and frequency can be computed from these instantaneous frequency metrics. A sparse matrix H∈R^(T × F) is filled with the instantaneous amplitudes value from the IMFs at their respective time and frequency co-ordinates. This matrix is the Hilbert-Huang Transform (HHT; See figure 2E for an example) and provides an alternative time-frequency transform to traditional Fourier based methods such as the short-time Fourier transform and the wavelet transform (15).

#### 2.2.2 Cycle Detection

The next stage is to segment the IMFs into their constituent cycles and identify which cycles will be included in further analysis. The start and end of theta cycles are located by the differentials greater than six in the phase. The start and end point of cycles in this paper is the ascending zero-crossing as this occurs at the point where the phase time-course wraps. Once identified, some cycles will be ‘bad’ in the sense that the oscillation captured by the IMF is not well represented, e.g., because the rhythm is not present over that time period or it is poorly estimated, and will be excluded from subsequent analyses. This is important for instantaneous frequency analyses as the differentiation step (see section 2.2.1) can be very noise sensitive. Included cycles are identified from the wrapped instantaneous phase time-course of the IMF containing the oscillation to be analysed. As the instantaneous phase computation via the Hilbert transform returns a value for every sample regardless of whether a prominent rhythm is present, only ‘good’ theta cycles are retained for further analysis. A good cycle is defined as having a phase with a strictly positive differential (i.e., no phase reversals) that starts with a value 0≤x≤π/24 and end within 2π-π/24≤x≤2π and 4 control points (peak, trough, ascending edge and descending edge).

#### 2.2.3 Control Point analysis

One approach to quantify oscillatory waveforms is compare the relative timings of the peak, trough, ascending zero-crossing and descending zero-crossing within each cycle (6, 7). These four control points can be straightforwardly identified in each cycle and used to summarise waveform shape by a range of different metrics. The ratio of temporal durations between these control points can describe large scale shape features. In the present analyses, the extrema (peaks and troughs) are detected by finding the samples closest to a zero-crossing in the differential of an IMF. It is unlikely that the peak or trough occur exactly coincident with timing of the sampling during data acquisition, so the extrema locations are refined to a point between samples using parabolic interpolation (34). The zero-crossings are initially identified from sign changes in the IMF time-course and refined by linear interpolation between the samples around the zero-crossing. Finally, we use these single cycle timings to compute the peak-to-trough ratio and the ascent-to-descent ratios for each cycle (7).

#### 2.2.4 Phase-alignment

We present an alternative approach to control points that ensures that entire waveform profiles can be combined and/or compared across cycles despite cycle-by-cycle differences in progression rate and overall duration. To compare waveforms across cycles that play out at different speeds, we use phase-alignment to register cycles onto a common grid. Phase alignment is performed on the instantaneous phase of a cycle and a measure of interest, such as the instantaneous frequency, which is observed at the same time-intervals. A linear one-dimensional interpolation function is fitted between the instantaneous phase (as x values) and the instantaneous frequency (as y values). The interpolation function is evaluated on a template set of instantaneous phase values with a linear spacing between 0 and 2pi, if any points in the template fall outside the fitted range, the interpolator returns an extrapolated value. This interpolated version of instantaneous frequency is then directly comparable across cycles as each point in the phase will occur at the same time. We compute phase-alignment using a linear interpolation across 48 fixed points across the zero to 2pi phase range.

Once an instantaneous frequency profile has been phase aligned, we can visualise a normalised waveform by projecting the frequency content back to a phase-time course. This is achieved by re-normalising the instantaneous frequency from Hertz back to radians in order to create a profile of successive phase differences. The phase time-course is then reconstructed from the cumulative summation of these phase differences. An oscillatory waveform can then be computed by taking the sine transform of this phase time-course. The resulting waveform has an amplitude of one and a consistent time-axis for all cycles. This ‘normalised waveform’ allows for visualisation of shape between cycles with different durations and amplitudes.

#### 2.2.5 Describing shape an instantaneous frequency mean vector

A simplified summary of a cycle’s shape can be computed from a mean-vector of the phase aligned instantaneous frequency according to the following equation:

$$\mathrm{Mean}\;\mathrm{Vector}=\bar{IF}e^{i\bar{\emptyset }}$$

Where $\bar{\emptyset }$ is the uniform phase-grid used in phase-alignment and $\bar{IF}$ is the phase-aligned instantaneous frequency. This is similar to the mean-vector approach to computing phase-amplitude-coupling (35). The mean-vector of a sinusoidal cycle will be zero whilst a non-sinusoidal cycle will return a complex value whose angle indicates which phase has the highest instantaneous frequency and the magnitude indicates the extent of the frequency modulation through the cycle. This method provides a straightforward summary but is only sensitive to unimodal deviations from a flat instantaneous frequency profile.

The mean vectors of many cycles can be visualised by a scatter plot in a 2-dimensional space. The real and imaginary parts of the complex valued mean-vector are plotted on the x and y-axes respectively. A sinusoidal cycle would have a mean vector of zero and appear at the origin on this space. Non-zero real values on the x-axis indicates the ascending to descending edge asymmetry in the cycle and non-zero imaginary values on the y-axis indicates peak to trough asymmetry.

#### 2.2.6 Principal Components shape motifs

The phase-aligned instantaneous frequency profiles provide a detailed description of each cycle’s shape, but additional analyses are required to identify any trends or consistencies across cycles within a dataset. Here, we look to take a data-driven approach to describe the main characteristics of oscillatory waveform in our simulated and real datasets.

We used Principal Components Analysis (PCA) to identify the principal modes of variation in shape across the included cycles. Phase-aligned cycles are concatenated into a single matrix of size [nphases x ncycles]. The second dimension of this matrix (across cycles) is reduced to PCA resulting in a [nphases x ncomponents] matrix of shape ‘motifs’. Each component motif is defined by the distribution of component weights across phase and an [ncomponents x ncycles] matrix of PC scores indicating the presence of each component motif in each individual cycle. The shape of each individual cycle can then be approximated by a linear combination of each shape motif weighted by the cycle’s component scores.

The PCA allows us to straightforwardly visualise the range of shapes within each dataset using the components and their dynamics over time using the scores. The shapes represented by each component motif can be visualised by defining a set of scores containing the maximum or minimum observed score for the PC in question and zeros for all others. These scores can be projected back into the original data space to provide exemplar instantaneous frequency profiles for both extremes of the PC axes. These exemplar instantaneous frequency profiles can be projected back into the time-domain to generate a normalised waveform that preserves the shape depicted in the exemplar instantaneous frequency profiles. The dynamics of each shape across cycles are represented by the PC scores. The relationship between these dynamics and other factors such as movement speed, theta amplitude and theta duration are quantified in the general linear model analysis described in section 2.4.4.

### 2.3 Simulation analyses

#### 2.3.1 Schematic cycle generation

To illustrate the relationship between waveform shape, instantaneous phase and frequency, a set of noise free oscillations were generated. First, a linearly progressing phase time-course is generated and sinusoid is created by taking a sine-transform of this wrapped phase. Different non-sinusoidal cycles are generated by modulating the unwrapped phase time-course by sine and cosine waves at different phases and frequencies. The cycles with extrema and edge asymmetry are generated by modulating the phase with a 1 Hz sine or cosine respectively. The extrema curvature examples are generated by modulating the linear phase with a 2 Hz sinusoid. From the computed cycle time-courses the instantaneous phase and instantaneous frequency are re-estimated using the Hilbert transform. Finally, the waveform shape is represented by phase-aligning the instantaneous frequency time-course of each cycle type with its instantaneous phase.

#### 2.3.2 Noisy Signal Generation

A more realistic noisy simulation was used for the results in figures 3, 4 and 5. A simulated oscillation at 12 Hz was generated using an autoregressive oscillator with the following transfer function:

$$H(f)=\frac{1}{1-2r\cos\theta +r^{2}}$$

Where *θ* is the angular frequency of the oscillator (in rads/sec) and *r* is the magnitude of the roots of the polynomial (0 < r < 1). For this simulation, we computed *H* for r=0.95 and *θ* equivalent to 12 Hz and used its parameters to filter (a forwards and backwards filter using scipy.signal.filtfilt) white noise. This generates a noisy sinusoidal oscillation which contains random dynamics in the frequency and amplitude of each oscillatory cycle. Sixty seconds of data were generated at 512 Hz.

This simulated oscillation was then modulated by one of two equations defined in equations 50.24 and 50.25 in section 50-6 in Volume 1 of Feynman’s Lectures of Physics (36). The first equation defines a linear system that scales the signal by a constant leaving the waveform shape unchanged.

$$x_{\mathrm{out}}=Kx_{\mathrm{in}}+e(t)$$

Whilst the second equation defines a nonlinear system that includes a term inducing a change in waveform shape as well as scaling the signal.

$$x_{\mathrm{out}}=K\left[x_{\mathrm{in}}+\epsilon x_{in}^{2}\right]+e(t)$$

The nonlinearity in the second equation makes the peak of the oscillations shorter and widens the trough. Both systems include an additive white noise term.

Finally, a signal with dynamic waveform shape changes was created. A dynamic oscillation was generated at 12Hz using the autoregressive oscillator method before phase of this signal was estimated using the Hilbert transform. Each cycle was then randomly assigned to a ‘sinusoidal’, ‘fast ascending or ‘fast descending’ category. The phase of the non-sinusoidal cycles was modulated using an additive sinusoid in the same manner as the schematic cycle generation in section 2.3.1. Additional gaussian noise was then added to this dynamically non-sinusoidal signal.

#### 2.3.3 Noisy Signal Analysis

The simulations are separated into IMFs using the masked sift. The relatively straightforward dynamics in this simulation allow a simplified mask-sift to be applied. To define the mask frequencies, a first IMF is extracted using the standard sift routine. The number of zero-crossings in this IMF defines the frequency of the initial mask with subsequent masks being applied at half the frequency of the previous one. The mask amplitudes are set equivalent to 1-standard deviation of the previously extracted IMF.

The 12-Hz oscillation is isolated in the third IMF (IMF-3) of this mask sift. The frequency transformation of this IMF is computed using the Hilbert transform (section 2.2.1). The Hilbert-Huang transform (HHT) is computed using 64 frequency bins between 2 and 35 Hz. A wavelet transform was computed using a 5-cycle Morlet basis computed using the same 64 frequencies as the HHT.

The timing of individual oscillatory cycles is identified using the phase jumps in the instantaneous phase time course where the oscillatory amplitude was above a threshold of 0.04 and instantaneous frequency below 18Hz. We compute control-point ratios (section 2.2.3), time locked instantaneous frequency profile and phase aligned instantaneous frequency profile (section 2.2.4) across all included cycles (see section 2.2.2) of IMF-3.

The sift, frequency transform, cycle detection and shape metrics are computed for both the static and dynamic systems defined in section 2.3.2. The control point metrics and phase-aligned instantaneous frequency profiles are computed from the static signal. Finally, the PCA shape motifs method is applied to the phase-aligned instantaneous frequency profiles of the cycles in the dynamic signal. Component scores, and normalised waveforms are computed from the first principal component. The component scores are then compared to the known cycle shape categories and with the ascending to descending asymmetry control point ratio.

### 2.4 Hippocampal theta analyses

#### 2.4.1 Animals

The data used here were obtained from the previous study by Lopes-dos-Santos et al. (2018) (20). Animals were male adult (4–7 months old) C57BL/6J mice (Charles River, UK) or transgenic heterozygous Cre-driver mice (Jackson Laboratories; obtained from C57BL/6J crossed with CamKIIa-Cre B6.Cg-Tg(Camk2a-cre)T29-1Stl/J, stock number 005359, RRID: IMSR_JAX:005359). All animals had free access to water and food in a dedicated housing facility with a 12-h light/12-h dark cycle (lights on at 7:00), 19–23 °C ambient temperature and 40–70% humidity. All mice were held in individually ventilated cages with wooden chew sticks and nestlets. The experimental procedures performed on these mice were conducted in accordance with the Animals (Scientific Procedures) Act, 1986 (United Kingdom), with final ethical review by the Animals in Science Regulation Unit of the UK Home Office.

#### 2.4.2 Local Field Potentials

Local field potentials (LFPs) were recorded from the pyramidal layer of hippocampal CA1 using multi-channel tetrodes (20). Recordings were made during open-field exploration in both familiar and novel environments across two recordings taken from each of three mice to make a total of six analysed datasets. Further data acquisition details can be found in (20) and the supplemental materials (Section 8; https://doi.org/10.6084/m9.figshare.15028986).

#### 2.4.3 Mask sift and Frequency transform

**Figure 1:**
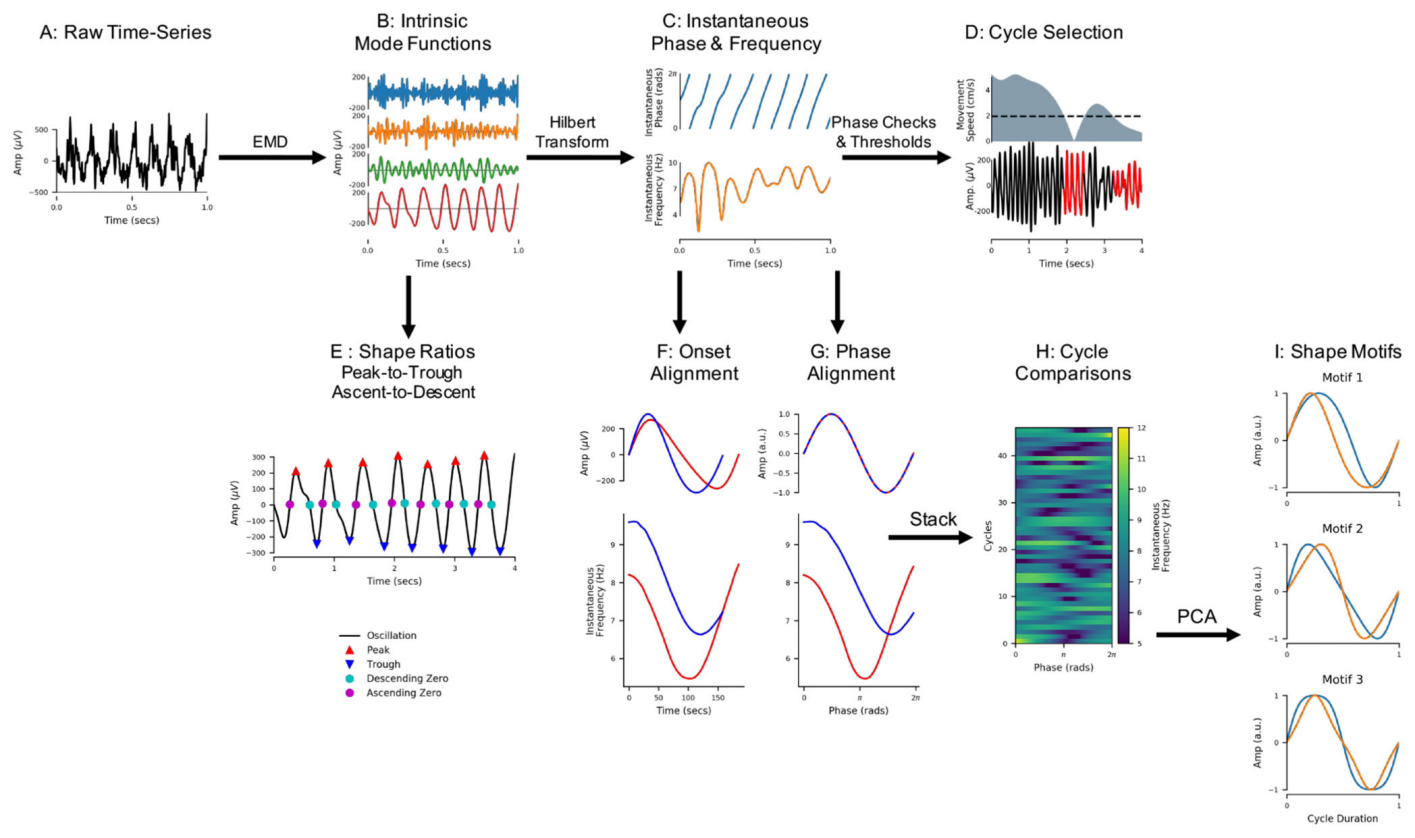
Overview of the analysis framework applied to LFP recordings of hippocampal theta. A: The raw input LFP recording. B: The raw signal is split into Intrinsic Mode Functions (IMFs) using a mask sift. C: An instantaneous phase and instantaneous frequency time-course is estimated from the theta IMF using the Hilbert Transform. D: Cycle start and stop times are identified from jumps in the wrapped phase time-course and ‘bad’ cycles with distortions of reversals in phase are identified and removed. E: Control points (peaks, troughs, ascending zero crossings and descending zero crossings) are estimated from the good cycles within the theta IMF. Shape is then summarised using peak-to-trough and ascending-to-descending duration ratios. F: The instantaneous frequency of each good cycle is phase aligned to correct for variability in cycle duration and internal cycle timings. G: The phase-aligned cycles are stacked into a single array to allow for straightforward comparisons between cycles. H: The phase-aligned instantaneous frequency of a group of cycles ready for comparison. I: A set of shape motifs are identified from the matrix in H using PCA.

LFP recordings were each separated into oscillatory components using the mask sift (Figure 1 A & B) with masks placed at f_m = [350, 200, 70, 40, 30, 7, 1 Hz]. These masks were selected to capture components with frequencies above the mask frequency down to around f_m*0.7 (37, 38). Keeping the masks constant across recordings ensures that the frequency content of each IMF will be comparable across recordings. For instance, we isolate the theta oscillation in IMF-6 using a mask frequency of 7 Hz. These mask frequency parameters were validated by rerunning the phase-aligned instantaneous frequency analyses with jittered mask-frequencies (Supplemental section 8.3; figure S3; https://doi.org/10.6084/m9.figshare.15028986), showing that the theta waveform description is robust to reasonable changes to the mask frequency values. These mask frequencies were effective in this set of CA1 LFP recordings, but it is expected that a different set of mask frequencies would be needed to analyse time-series containing different oscillatory dynamics. Next, a frequency transformation was computed for each IMF using the Hilbert transform and the methods from section 2.2.1 (Figure 1C).

#### 2.4.4 Cycle detection

To ensure that the detected theta cycles are physiologically interpretable theta activity, we identified cycles in each recording during times where the speed of movement of the mouse was greater than 1 cm/second. As faster movement is associated with stronger theta oscillations, this restriction increases the probability that our detected cycles represent physiologically interpretable theta events. We additionally restricted analyses to cycles in IMF-6 where cycle duration corresponded to 4-11 Hz frequency range (i.e., 312 and 113 samples respectively) and cycle amplitude was above the bottom 10% of the amplitude distribution. Finally, cycles that failed the cycle inclusion checks outlined in section 2.2.2 were removed from analysis at this point (Figure 1D).

#### 2.4.5 Cycle comparisons

We computed the temporally aligned instantaneous frequency profile, phase-aligned instantaneous frequency profile and normalised waveform for each included theta cycle. The average waveform shape within each dataset was estimated from the averaged phase aligned instantaneous frequency (Figure 1F & G) and a group average constructed from the mean of the six individual runs. Variability in waveform shape across single cycles in the group data is summarised using the instantaneous frequency mean vector (section 2.2.5) and visualised as a distribution in the complex plane in which the x-axis represents asymmetry between ascending and descending edge frequency and the y-axis represents asymmetry between peak and tough frequency. For comparison, we also identified the control points from each cycle of the theta IMF and constructed the peak-to-trough and ascending-to-descending duration ratios (7).

#### 2.4.6 Waveform Motifs and relation to behaviour

We next look to explore the waveform shapes which are present in the phase-aligned instantaneous frequency values. We use PCA (section 2.2.6) to identify the data-driven set of shape components that explain the most variance in the shape of theta cycles in this dataset (figure 1H). The first four principal components explaining 95% of variance defined our four shape motifs and were retained for further analysis. The reproducibility of the PCA is validated across 500 split-half iterations assessing the proportion of variance explained by each PC and the correspondence between the component shape in the two halves (Supplemental section 8.3; figure S3).

We next use a General Linear Model (GLM) to quantify how between cycle variability in the shape motifs relates to other behavioural and electrophysiological covariates (i.e., cycle amplitude, cycle duration and mouse movement speed). The GLM was created with a design matrix containing the mean and the three z-transformed covariates. These predictors were used to model the between cycle variability in the principal component (PC) scores for each shape component in turn. This resulted in four GLMs each fitting four parameter estimates, one mean term and three parametric effects. The GLM parameters were fitted using a Ordinary Least Squares algorithm implemented in python (https://pypi.org/project/glmtools/). The t-statistic of each parameter estimate was computed, and statistical significance established using a row-shuffle non-parametric permutation scheme. Each of the three parametric regressors was permuted separately, only shuffling values within the regressor of interest for that permutation whilst keeping the covariate structure constant. 5000 permutations were computed for each PC-motif and dependent variable before the threshold for statistical significance was determined at p<0.01. The observed t-statistics from the un-shuffled data was then compared to this critical value to identify which effects could be considered statistically significant.

## 3 Results

### 3.1 Instantaneous Frequency tracks waveform shape

**Figure 2:**
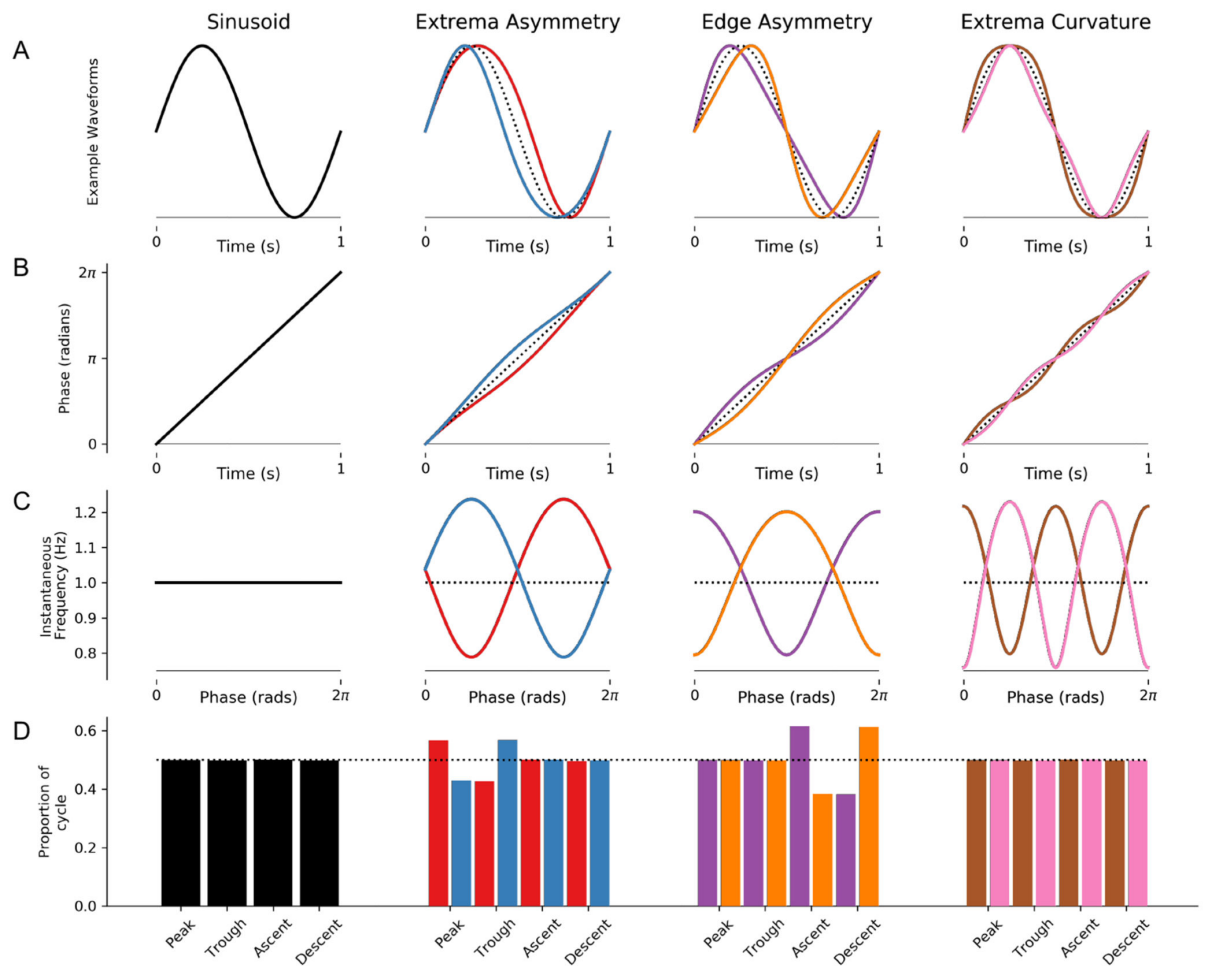
Instantaneous Frequency changes with oscillatory waveform shape. The four columns illustrate examples of different simulated oscillatory cycles with distinct waveform shapes. A: The time-domain waveforms for each cycle. The first column shows a sinusoid, and the remaining three columns show pairs of cycles with opposite waveform distortions (for reference a sinusoid is shown as a dotted back line). B: The instantaneous phase time course of the signals in the corresponding column. C: The instantaneous frequency time course of the signals in the corresponding column. D: The durations between different control points for each cycle the dotted line indicates the expected duration for a sinusoid.

Figure 2 illustrates how instantaneous frequency reflects waveform shape in a set of noiseless simulated cycles (see section 2.3.1). A sinusoidal cycle (Figure 2A) has a monotonically progressing phase time-course which, in turn, has a flat instantaneous frequency profile. Analysis of the duration of different segments reveals that the peak, trough, ascending edge and descending edge all have the same duration. Cycles with a narrow peak, trough or descending edge show corresponding changes in their instantaneous frequency (Figure2 B & C). Specifically, the longer duration, slower features correspond to a lower instantaneous frequency. These instantaneous frequency profiles can describe a wide range of possible shapes. For example, cycles in which both the peak and trough are widened or pinched lead to instantaneous frequency profiles with multiple extrema (Figure 2: last column). While the simple control-point metrics used here can track individual waveform features such as peak or trough duration (Figure 2: bottom row), the quantification of more complex shapes would require the definition of additional control points and shape metrics.

### 3.2 Quantifying and comparing waveform shape in a simulated signal

**Figure 3:**
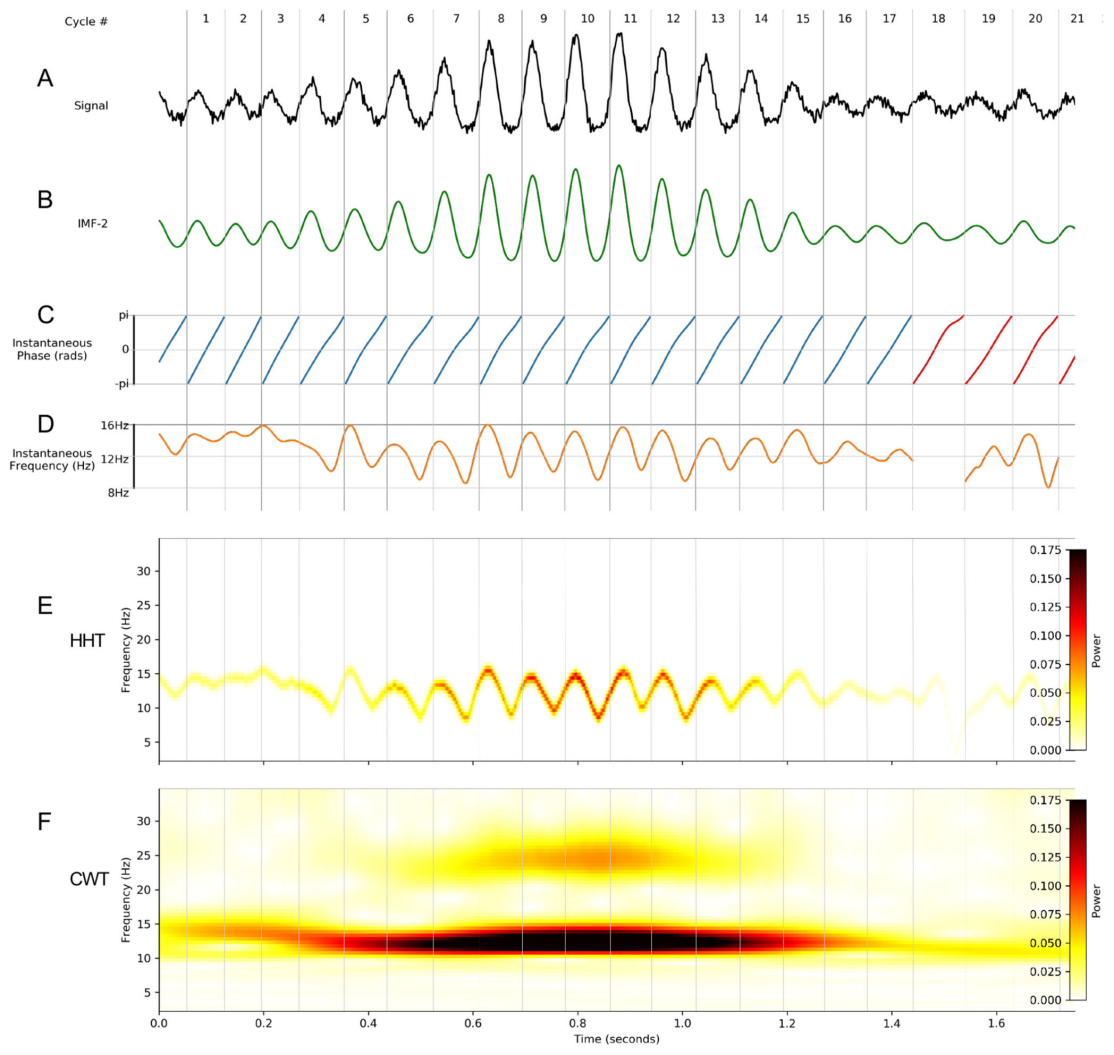
Instantaneous frequency analysis on a noisy non-sinusoidal oscillation. A simulated 12 Hz oscillation can be extracted from a noisy time-series and represented with EMD, instantaneous frequency and the Hilbert-Huang Transform. Vertical grey lines denote the starting times of individual cycles across the different panels. A: The simulated noisy non-sinusoidal oscillation. B: IMF-3 extracted from ‘A’ containing the simulated oscillation. C: Instantaneous phase time-course of IMF-3. Cycles excluded from further analysis are indicated in red. In this case, these cycles were below the amplitude threshold. D: Instantaneous frequency time-course of IMF-3. E: Hilbert-Huang Transform (HHT) of the simulated data segment. F: Continuous Wavelet Transform (CWT) of the simulated data segment.

We next use simulations to illustrate how instantaneous frequency analyses can be conducted on a noisy signal with a dynamic 12 Hz oscillation modified by a non-linearity which widens the trough of each cycle (see section 2.3.2). This oscillation was isolated from the noisy background using mask sift (Figure 3B) before the Hilbert transform was used to compute the instantaneous phase time-course (Figure 3C). It is evident that the phase time-courses do not progress linearly through all cycles; these deviations from monotonic phase progression are quantified in the instantaneous frequency time-course (Figure 3D). Instantaneous frequency sweeps within a single cycle reflect the non-sinusoidal shapes of the time-domain waveforms. For this simulation, the instantaneous frequency tends to be higher during the first half of the cycle and lower in the second half, reflecting the non-linearity that shortens the peak and widens the trough of these oscillations.

The HHT of the simulated signal (Figure 3E) retains the high time-frequency resolution of the instantaneous frequency time-course allowing within cycle frequency dynamics to be visible. In contrast, while a standard 5-cycle Morlet wavelet transform identifies similar power dynamics, variability in frequency within single cycles are not resolved (Figure 3F). A further disadvantage is that the non-sinusoidal waveform shape of this simulation introduces a 24Hz harmonic component into the wavelet transform.

**Figure 4:**
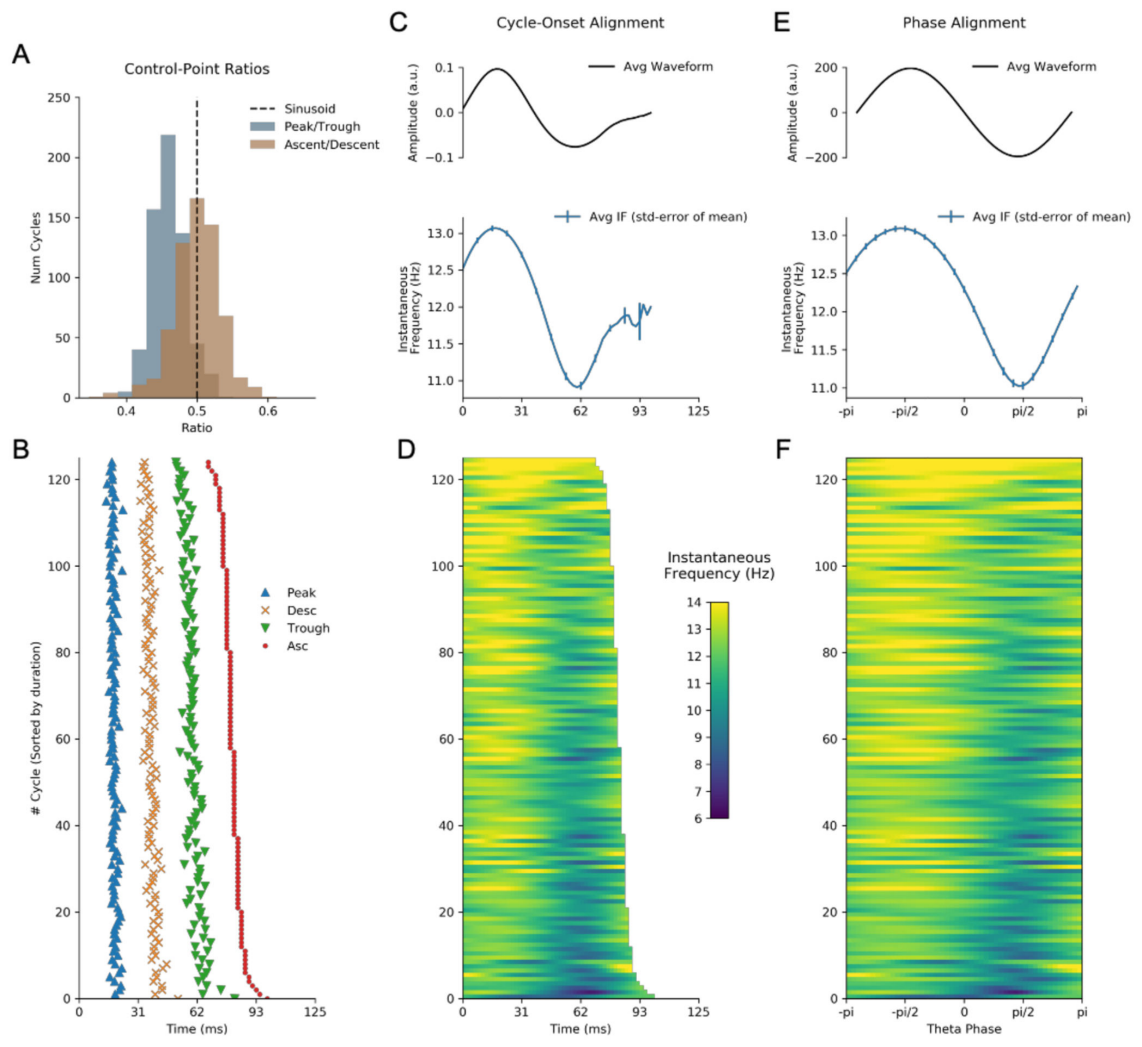
Methods for comparing waveform across cycles. Simulated data illustrating how waveform shape can be quantified using control point ratios, instantaneous frequency and phase-alignment. A : Distribution of the peak-to-trough and ascent-to-descent ratios for the simulated dataset. B : The timing of the control points used to create the ratios in A for an illustrative subset of the cycles C : Top: Average temporally aligned signal waveform. Bottom: Average temporally aligned instantaneous frequency profiles. D : Illustration of single cycle temporally aligned instantaneous frequency profiles for an illustrative subset of the cycles. E : Top: Average phase aligned signal waveform. Bottom: Average phase aligned instantaneous frequency profile. F : Illustration of single cycle phase aligned instantaneous frequency profiles for an illustrative subset of the cycles.

Individual cycles of an oscillation play out at different rates leading to differences in the timing of extrema within cycles and in overall cycle duration. These two sources of variability hamper comparisons between individual oscillatory cycles. As outlined above (section 2.2.3) one method to solve this issue is to discretise the cycle using a set of control points before computing the proportion of time spent in different segments of the cycle (6, 7). The cycle phase quartiles (ascending zero-crossing, peak, descending zero-crossing and trough) of 500 cycles of the simulated signal is shown in Figure 4A. The ratio between peak-to-trough duration suggests that these cycles have relatively long troughs, whilst the ratio between ascending-to-descending duration suggests that the rising and falling phases are approximately equal in duration (Figure 4B).

An alternative approach for comparing cycles is to align the instantaneous frequency profiles to one of the control points. For example, we aligned the 500 cycles to the ascending zero-crossing and computed their time-locked average (Figure 4C). The time-locked instantaneous frequency profile of these cycles is not flat, reflecting the presence of non-sinusoidal shape in this simulated signal. However, the precise type of non-sinusoidal shape is ambiguous from this average, due to variability in the location of different waveform features within single cycles. In this case, the instantaneous frequency is highest around 10 samples after the ascending zero-crossing; however, this time-lag might correspond to different points in the waveform in different cycles. In addition, variability in the duration of cycles means that, after a certain point, different numbers of cycles contribute to the average, making the estimate unstable.

Here we present an approach that overcomes these shortcomings. In brief, phase-alignment removes this ambiguity by visualising the instantaneous frequency of a cycle across a fixed grid of points along its phase (see section 2.2.3). For instance, an oscillatory peak is normalised to occur at the same phase value irrespective of the cycle’s duration or shape. This corresponds always to exactly one quarter of the phase of each cycle, but not necessarily to one quarter of the duration of each cycle. By aligning the instantaneous frequency to the phase, we remove the temporal distortions caused by varying shapes and cycle durations, and express the shape with the phase-aligned instantaneous frequency values. The phase-aligned instantaneous frequency of the simulated cycles (Figure 4D) now unambiguously shows the increased frequency around the peak of the 12Hz oscillation and decreased frequency around the trough. The average across the phase-aligned cycles is then a smooth representation of the shape of the entire cycle.

**Figure 5:**
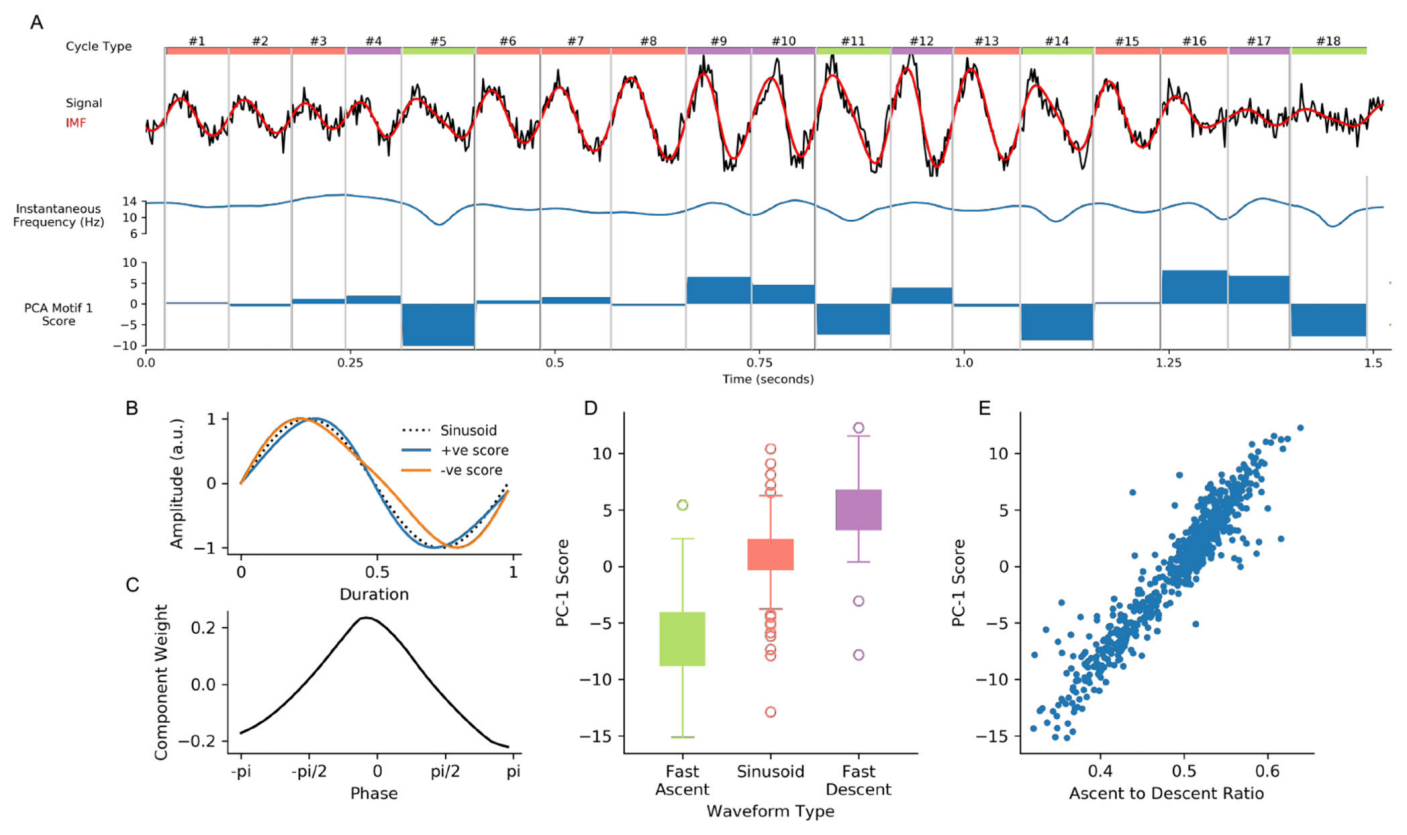
Quantifying dynamics in waveform shape. A : A simulated oscillation is contains cycles that are sinusoidal or contain a ascending to descending edge asymmetry. The raw signal is shown (black line) with the EMD extracted IMF (red) and instantaneous frequency (blue). The PC component score for each cycle is show in the bar plot. B : Normalised waveforms for principal component 1 of the simulated data. This shows that the component captures a continuum between a fast ascending and a fast-descending shape C : The component shape for component 1 showing that the component score leads to a difference in instantaneous frequency between the ascending and descending phase of the cycle. D : The component score distribution for each of the know shape categories. E : The relationship between the data-drive principal component score and the a priori defined ascent to descent ratio.

Comparisons between sets of cycles is straightforward once waveform shape has been estimated from instantaneous frequency and normalised through phase-alignment. This is illustrated in a simulation containing dynamic changes in oscillatory waveform shape (section 2.3.2; Figure 5A). Differences in the ascending and descending durations can be seen between cycles in both the raw time-course and the extracted IMF. Modulations in instantaneous frequency track these dynamics. A principal components analysis of the phase-aligned instantaneous frequency profiles of 60 seconds of simulated data is able to quantify these dynamics. The component score clearly tracks the differences in waveform, with fast ascending cycles having large negative scores, fast descending cycles having large positive scores and sinusoids having scores close to zero. The normalised waveform and component shape confirm that the first component of this analysis represents a shape dimension ranging between fast ascending and fast descending shapes (Figure 5 B and C). The PC score varies strongly between the three shape categories (Figure 5D) and shows a very close correspondence to the more targeted ascending to descending duration control point ratio (Pearson’s r= 0.945, Figure 5E). This demonstrates that the phase-aligned instantaneous frequency profiles can distinguish different waveform shapes in cycles with rapidly changing shapes. Moreover, it is able to do so in a data-drive way, without pre-specification of the potential shape of interest.

### 3.3 Characterising waveform shape in hippocampal theta

**Figure 6:**
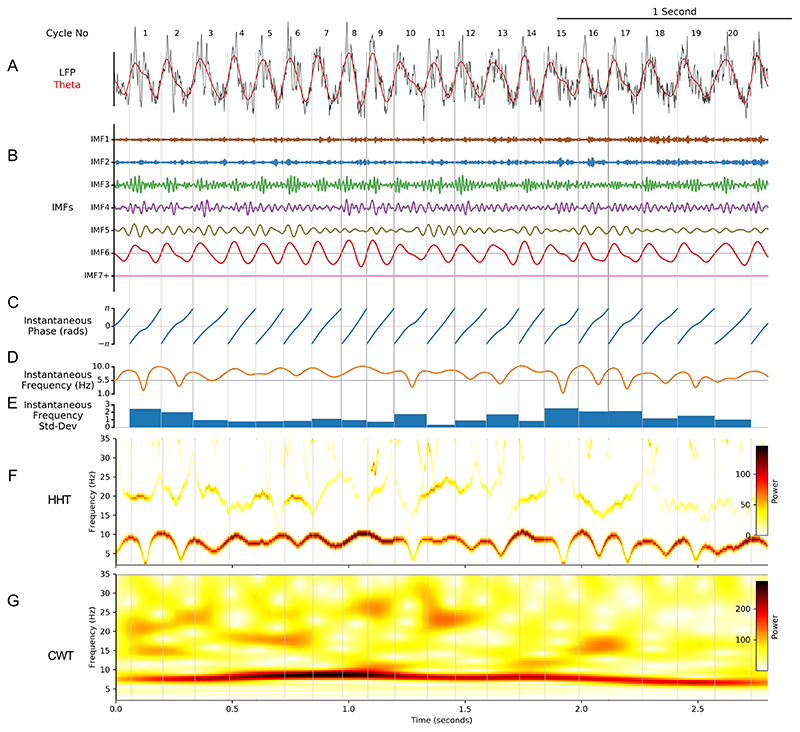
EMD analysis of a LFP segment containing hippocampal theta oscillations. A : A segment of a hippocampal LFP recording (black) overlaid with the extracted theta oscillation (red). B : IMFs extracted from this data segment using the mask-EMD. The theta oscillation is isolated into the IMF-6. C : Instantaneous phase time-course of the theta IMF. D : Instantaneous frequency time-course of the theta IMF. E : Variability in instantaneous frequency for each theta cycle. F : Hilbert-Huang Transform (HHT) of the LFP segment. G : Continuous Wavelet Transform (CWT) of the LFP segment.

LFP data recorded from the mouse hippocampus were analysed to explore the utility of phase-aligned instantaneous frequency as a measure of waveform shape. figure 6A shows a three-second LFP recording from the pyramidal layer of the mouse dorsal CA1 (black) overlaid with the EMD-extracted theta IMF (red, Figure 6B shows all IMFs). In this case, the theta oscillation was isolated into IMF 6 with minimal disruption to its amplitude or waveform shape dynamics. Many of the theta cycles within this window have prominent non-sinusoidal waveform shapes, which are qualitatively visible in both the raw data trace (24, 39) and the EMD-extracted theta IMF. Importantly, the oscillatory waveform shape varies between successive cycles, though the amplitude and duration of the theta cycles are relatively consistent.

The instantaneous phase (Figure 6C) and instantaneous frequency (Figure 6D) were computed from the theta oscillation in IMF-6. As with the simulation analysis, any within-cycle dynamics in the instantaneous frequency naturally represent the waveform of each cycle. This was summarised with the standard deviation of instantaneous frequency values within each cycle (Figure 6E). As an illustration, cycles 5, 9 and 11 have relatively sinusoidal shapes with flat instantaneous frequency profiles and low frequency variability. In contrast, cycle 13 is relatively non-sinusoidal with a dynamic instantaneous frequency profile and high frequency variability. The HHT provides a time-frequency description with sufficient resolution to depict these within cycle instantaneous frequency sweeps (Figure 6F). In contrast, a 5-cycle Morlet wavelet transform of the same data was not able to resolve these dynamics (Figure 6G). Note that, though some 20Hz power is present in the Hilbert-Huang Transform, this reflects oscillatory activity with distinct extrema from the theta base signal (see Supplemental section 8.4; Figure S4; https://doi.org/10.6084/m9.figshare.15028986)

**Figure 7:**
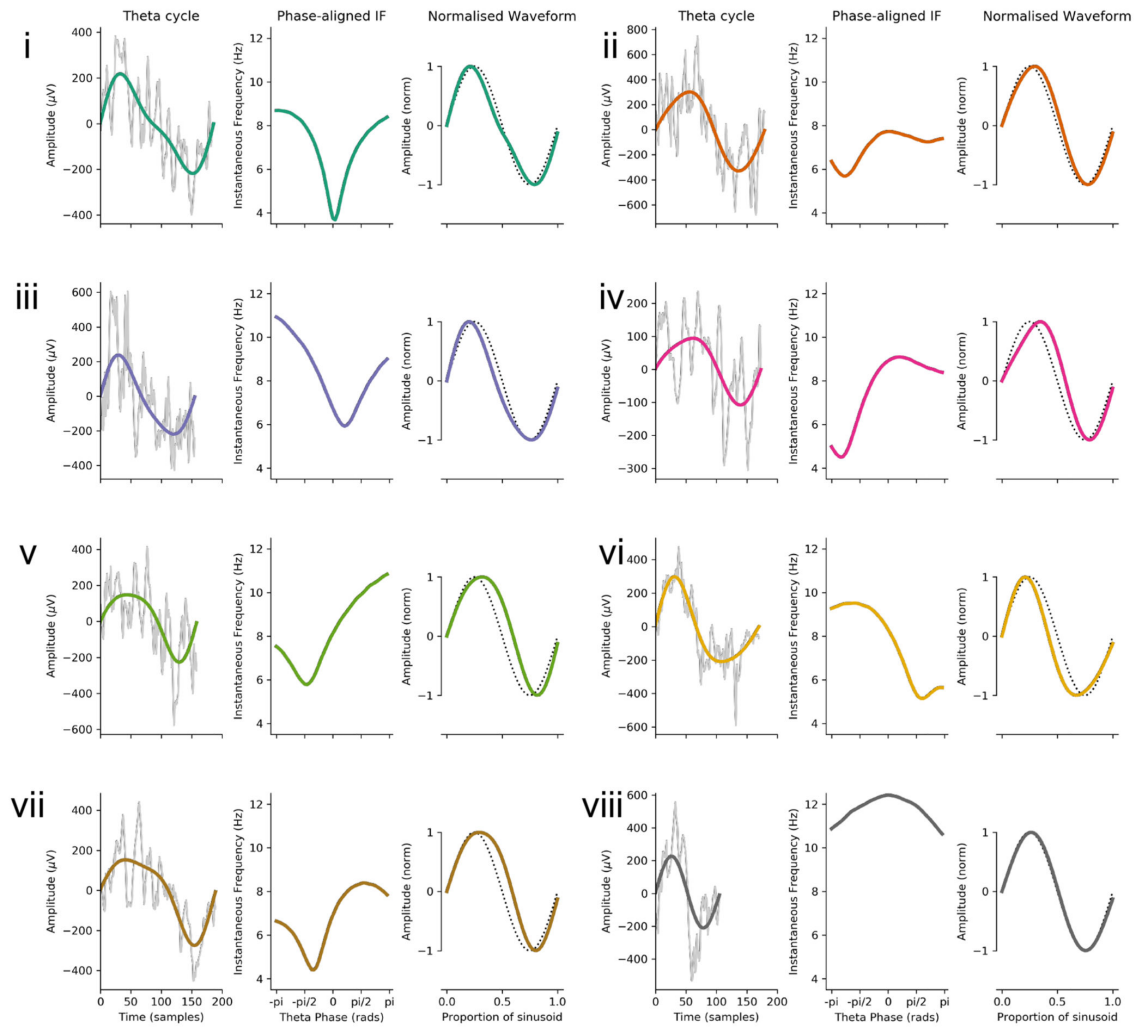
Characterising shape in eight example cycles of hippocampal theta. Eight representative theta cycles. For each example, the first subpanel shows the raw data (grey) with the theta IMF super imposed (coloured line). The second subpanel shows the phase aligned instantaneous frequency and third panel shows the normalised waveform (coloured line) with a sinusoid for reference (black dotted line).

Looking at individual cycles illustrates how instantaneous frequency can characterise waveform shape (Figure 7). Frequency increases and decreases correspond to slowing down and speeding up of the cycle as its waveform shows non-sinusoidal behaviour. It is evident that there are many observed shape profiles. For instance, the cycles labelled as *i* and *iii* in figure 7 had frequencies that dip during the centre of the cycle, indicating an elongated, low frequency descending edge. Cycle *v* had slowest frequency around -pi/2 corresponding to a wide peak. In contrast, cycle *vi* had relatively high frequency around -pi/2 and lower frequency at +pi/2 leading to a short, pinched peak and an elongated trough. Overall, the phase-aligned instantaneous frequency profiles and normalised waveforms provide a rich description of oscillatory waveform, despite wide variability in cycle amplitude, duration and shape.

### 3.4 Using phase-aligned instantaneous frequency to compare cycles

**Figure 8:**
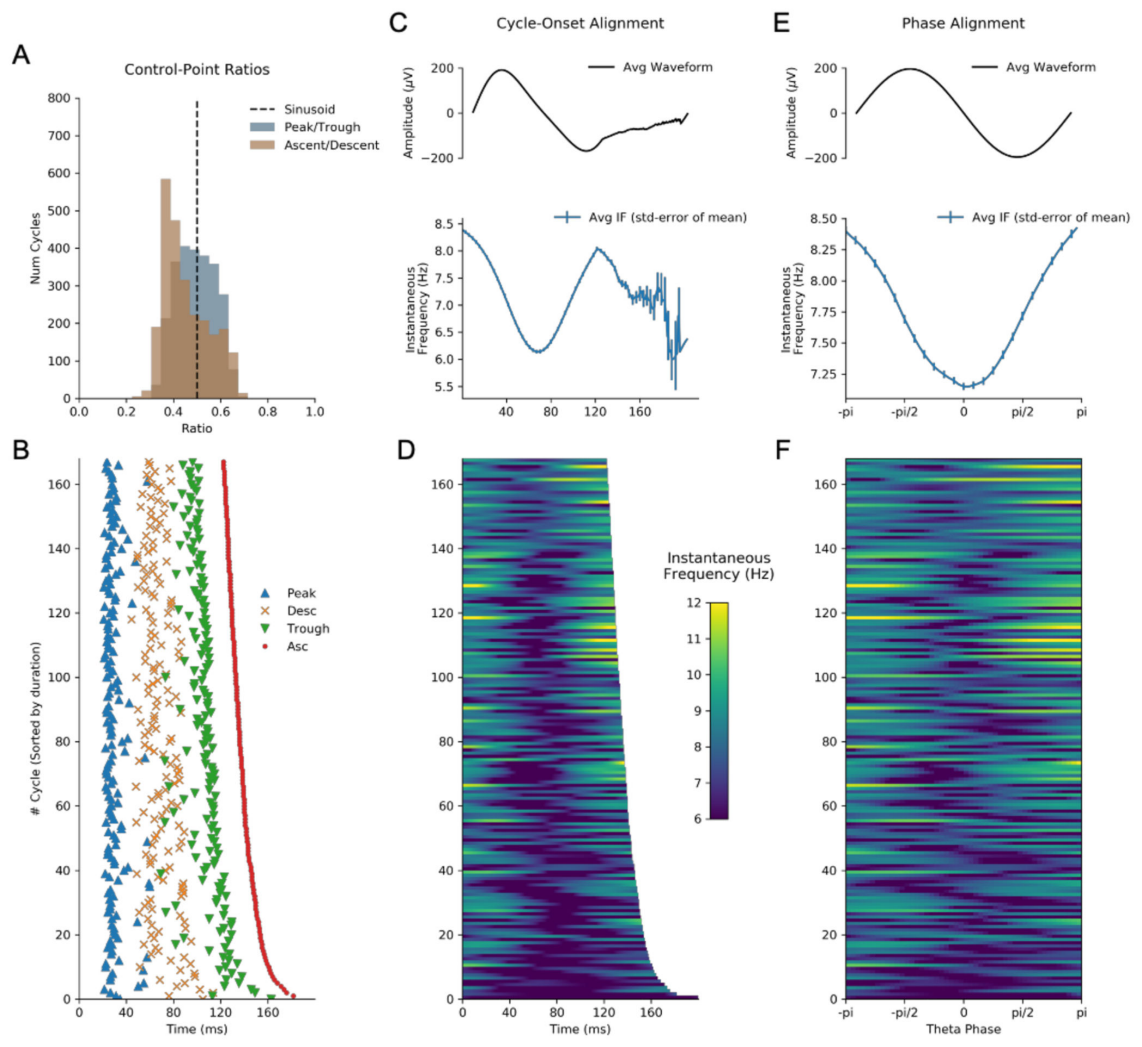
Methods for quantifying waveform in hippocampal theta. A : Distribution of the peak-to-trough and ascent-to-descent ratios for a single data recording. B : The timing of the control points used to create the ratios in A for an illustrative subset of the cycles C : Top: Average temporally aligned signal waveform. Bottom: Average temporally aligned instantaneous frequency profiles. D : Illustration of single cycle temporally aligned instantaneous frequency profiles for an illustrative subset of the cycles. E : Top: Average phase aligned signal waveform. Bottom: Average phase0aligned instantaneous frequency profile. F : Illustration of single cycle phase aligned instantaneous frequency profiles for an illustrative subset of the cycles.

We next computed waveform shape from around 1500 hippocampal theta cycles from a single recording using three different methods: control-point ratios (see section 2.2.6), control-point locking, and phase-alignment (see section 2.2.3). The durations between specified control points were computed for each cycle (Figure 8A) and the peak-to-trough ratio and the ascent-to-descent ratio computed (Figure 8B). A pair of one-sample t-tests against a mean value of 0.5 was used to assess whether the mean of these ratios significantly different from the value of a sinusoid. The peak-to-trough ratios (mean=0.49, standard deviation=0.08) showed a small shift towards a wider peak value, t(2513)=-2.66, p=0.008. The ascent-to-descent ratios (mean=0.45, standard deviation=0.096) showed a substantial shift towards longer descending cycles, t(2513)=-24.12, p<0.0001. The instantaneous frequency profiles locked to the ascending zero-crossing show a wide variety of shapes with a group average tendency for frequency to start around 9 Hz and to decrease through the duration of the cycle (Figure 8C). As described above, this average effect is challenging to interpret due to within-cycle variability in the timing of cycle features and between cycle variability in total cycle duration. Our proposed phase-aligned instantaneous frequency profiles (Figure 8D) resolve these ambiguities. This shows that theta cycle instantaneous frequency in this single recording starts around 9 Hz at the ascending zero-crossing, decreasing to around 8.1 Hz at the descending zero-crossing, before increasing again to 9 Hz at the end of the cycle. This is consistent with a fast-ascending and slow descending cycle shape revealed by the control point analysis and in previous literature. The phase-aligned instantaneous frequency approach is able to show this effect as a continuous shape profile for single cycles, which can be straightforwardly compared at the group level.

### 3.5 Theta has a stereotyped asymmetric shape with wide variability across cycles

**Figure 9:**
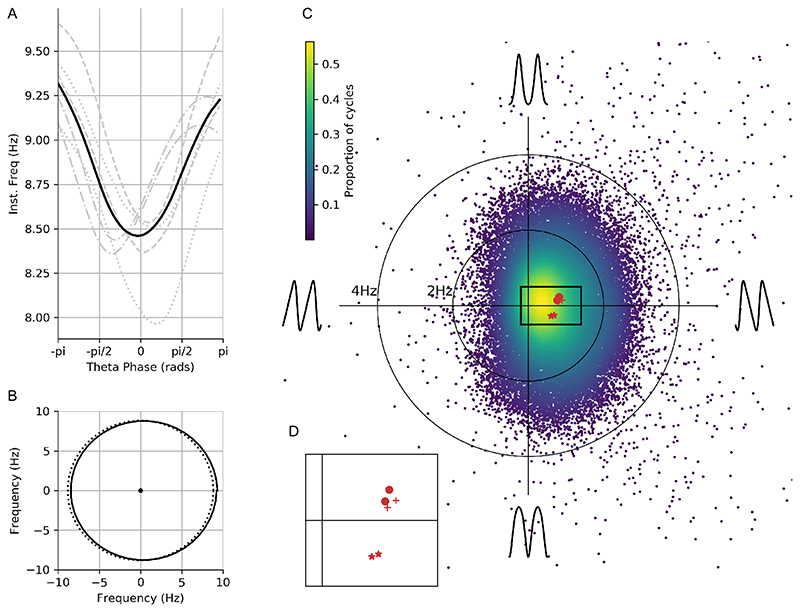
Average waveform shape and variability in shape across cycles in hippocampal theta. A : Average of phase-aligned instantaneous frequency profiles for each of the six separate recording sessions across three mice. The different dashed line styles indicate the runs from the different mice and the solid black line represents the average across all six recordings. B : Projection of the mean frequency profile from A around the unit circle (black line) with an equivalent projection of a sinusoid (dotted line). The mean frequency profile is clearly shifted to the right, indicating that the instantaneous frequency profile is not uniform across phase. The centre of this distribution is the mean vector whose magnitude represents the amount of frequency distortion across a cycle and whose angle represents where in the cycle the frequency is highest. C : The distribution of mean vectors for all individual cycles projected into a simplified ‘shape-space’ (see methods section 2.2.5) to visualise the overall variability in waveform shape around the average. Individual recording averages are shown in red with different symbols representing the three animals. D : Zoomed in section of 9C showing the individual recording sessions means.

We next summarised the average waveform across theta cycles from six recordings taken from three mice. The average phase-aligned instantaneous-frequency profile is computed for each recording and for the whole dataset. The overall group-level average waveform had a cosine-type profile centred around an average instantaneous frequency of ~8.6 Hz (Figure 9A, average in black and individual recording sessions in grey). On average, the instantaneous frequency peaked within the cycle around 9 Hz at the ascending zero-crossing and drops to just below 8.4 Hz between the peak and descending zero-crossing. These results are consistent with previous studies showing an asymmetry between the fast-rising and slow-decaying halves of a theta cycle (6, 7, 39). All six recordings across three animals showed a shape with a maximum frequency around the ascending zero-crossing and a minimum on the descending edge, though there was some variability in whether the lowest frequency was closer to the peak or trough.

To visualise the variability in waveform shape across cycles and recording sessions, we performed a complementary analysis using the instantaneous frequency mean-vector. The instantaneous frequency profile of each cycle is wrapped around the unit circle. The grand average profile shows a clear shift towards positive values on the x-axis which can be summarised by the mean value of the circular profile (Figure 9B). This can be repeated for every individual cycle to describe the distribution of single-cycle waveforms across a simplified 2-dimensional shape-space (Figure 9C; section 2.2.5). The x-axis of this space represents the amount of ascending to descending edge asymmetry in each cycle whilst the y-axis represents the amount of peak to trough asymmetry. One-sample t-tests against zero were performed on the x-axis and y-axis distributions to establish whether the overall distribution has a non-zero mean. The y-axis distribution, representing peak to trough asymmetry, was closely centred around the origin (mean=0.0025, standard deviation=0.92) with a mean that not significantly different from zero, t(40346)=0.55, p=0.58. In contrast, the x-axis distribution, representing ascending to descending edge asymmetry, has a larger, positive valued center (mean=0.38, standard deviation=0.63) which was significantly different to zero, t(40346)=122.75, p<0.0001. Overall, this indicates that the highest frequencies in a cycle are typically at the ascending edge, consistent with the average in Figure 9A and with previous literature on the theta cycle (6). Though the overall mean shift in the distribution of cycles is robust across recordings (Figure 9D), there is substantial cycle-to-cycle variability indicated by the width of the distribution.

### 3.6 Distinct waveform motifs are differentially related to behavioural and electrophysiological states

**Figure 10:**
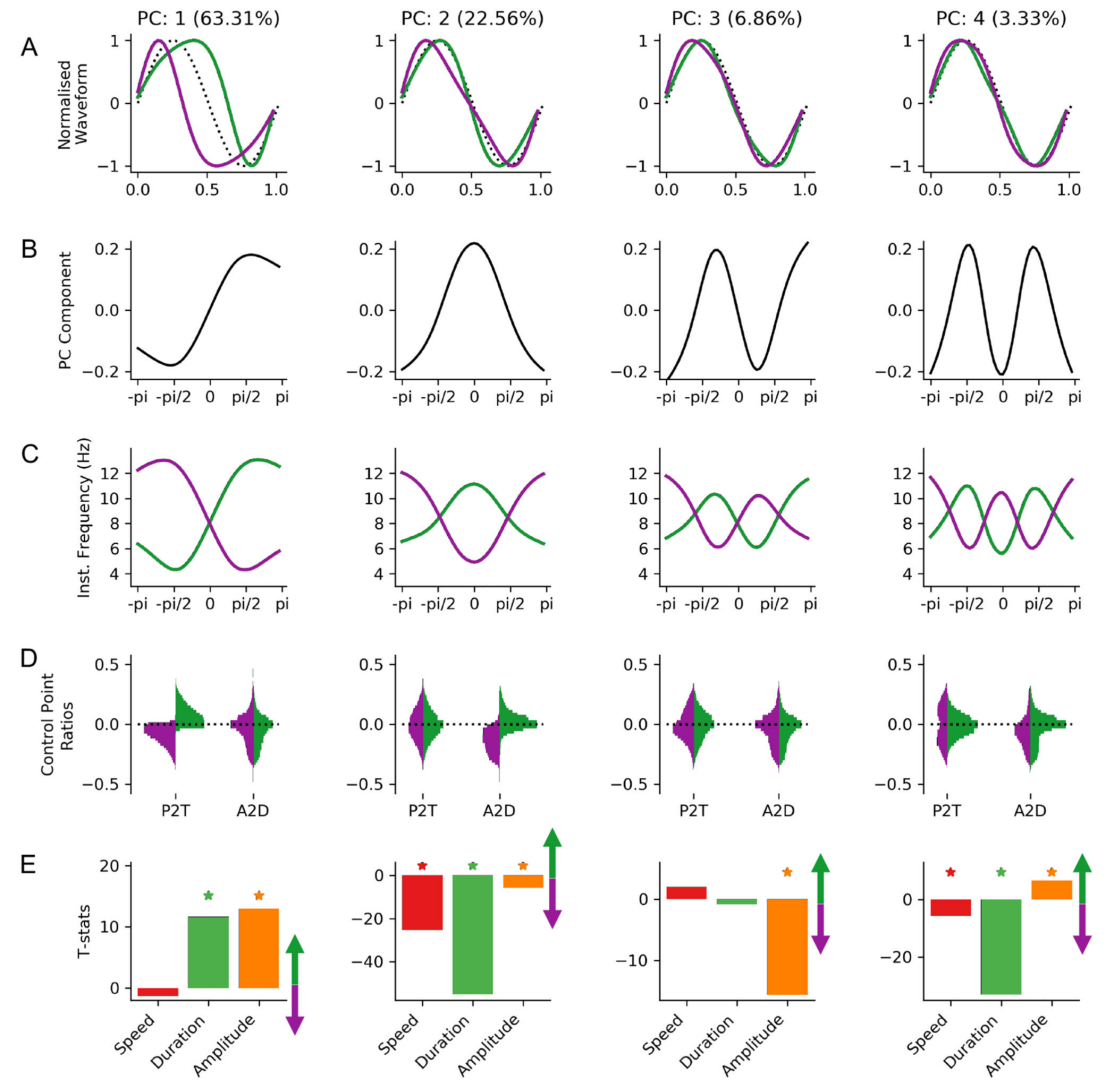
Shape motifs in hippocampal theta and their relation to movement speed. A : The normalised waveforms for the first four shape motifs identified from a PCA across all phase-aligned instantaneous frequency profiles. Waveforms for positive PC scores are shown in purple and waveforms for negative scores shown in green with a sinusoid for reference (black dotted line). B : PC for each motif. C : Instantaneous frequency profiles of each shape motif created by multiplying the PC shape in ‘B’ with the maximum or minimum observed PC score for that PC and adding the mean. Purple profiles represent the positive end of the score distribution and green profiles represent the negative end. D : Control-point ratios for cycles split by the sign of the PC score. Purple profiles represent the positive end of the score distribution and green profiles represent the negative end. E : t-value of a GLM modelling the PC score for each motif as a function of movement speed, theta cycle duration and theta cycle amplitude. Asterisks indicate statistical significance at p>0.01 as identified by non-parametric permutations.

To further describe the variability in waveform shape across cycles and characterise its relation to movement speed, theta amplitude and theta cycle duration we identify a set of waveform shape ‘motifs’ using PCA. The parameters of the PCA allow us to summarise the range of waveform shapes seen in the dataset from the components and the between cycle dynamics in shape from the component scores (see section 2.2.5 and 2.4.6). The robustness of the decomposition to random split-half subsampling was empirically assessed (See supplemental section 8.5; Figure S5; https://doi.org/10.6084/m9.figshare.15028986) The first four components describing 96% of variance are retained for further analysis (Figure 10). The shape of individual waveform motifs are visualised by their normalised waveforms (Figure 10A). These normalised waveforms are computed from the instantaneous frequency profiles of the PC component-vectors (Figure 10B) projected onto the extreme ends of the PC score distribution (Figure 10C). Each normalised waveform then describes one end of a distributions of shapes with all variability in amplitude and total cycle duration removed. The between cycle dynamics in shape can then be described by a set of four PC scores which describe the extent to which each shape component is expressed within each individual cycle.

PC-1 (63.31% of variance) describes a continuum of shape from a sharp peak and wide trough through to a wide peak and sharp trough. This shape is similar to the y-axis in the mean vector distribution in Figure 9B. In contrast, PC-2 (22.56% of variance) describes shapes ranging between an elongated ascending edge and an elongated descending edge, similar to the x-axis of Figure 9B. The remaining components describe more complex shapes with relatively small contributions to the variance explained. PC-3 (6.86% of variance) captures shapes with a left or right ‘tilt’ around their extrema and PC-4 (3.33% of variance) describes shapes with a sharper or flatter curvature around the extrema.

The control-point-based ascending-to-descending ratio and peak-to-trough ratio are computed for each cycle. For each PC, these values are partitioned into cycles with positive or negative PC scores (relating to distinct ends of the shape continuum for that component) and their distributions plotted in Figure 10D. The peak-to-trough ratios are clearly separated in the two ends of PC-1 whilst the ascending-to-descending ratios are similar for cycles with a positive or negative score in PC-1. This is consistent with the normalised waveforms summarising PC-1 in Figure 10A. PC-2 also shows the expected separation of ascending-to-descending ratios by PC score, whilst the peak-to-trough ratios are unchanged. Whilst PC-3 and PC-4 describe around 10% of shape variability, they are not characterised by the control point analyses. Neither peak-to-trough ratios nor ascending-to-descending ratios are changed by PC score for PC-3 or PC-4. These shape profiles are robustly identified by the phase-aligned instantaneous frequency method but are not distinguished by these control point-based metrics as the shape distortions in PC-3 and PC-4 occur between the four specified control points.

A general linear model was used to quantify the relationship between the different shape motifs and theta amplitude, theta duration and mouse movement speed. This regression is computed separately for each PC and the resulting parameter estimates converted into t-statistics. PC-1 codes for changes in average instantaneous frequency across the cycle with a small shape distortion around the descending edge. This PC has a strong relationship with cycle duration and amplitude but no significant covariation with movement speed. Longer and higher amplitude cycles tend to have more positive scores in PC-1 relating to wide peak shapes. PC-2 has a strong relationship with duration and movement speed. Specifically, cycles with elongated descending edges have longer cycle durations and are more likely to occur during fast animal movement. PC-3 shows significant covariance with cycle amplitude. High amplitude cycles tend to have shapes in which instantaneous frequency is relatively high just before the peak or trough. Finally, PC-4 varies strongly with duration and weakly with movement speed. Cycles with flatter curvatures around the extrema have longer cycle durations and are less likely to occur during faster animal movement.

## 4 Discussion

Non-sinusoidal waveforms are often visible by eye in raw LFP traces of electrophysiological datasets, yet discovering and quantifying these non-sinusoidal and non-linear features present substantial analytic challenges. We utilise within-cycle variability in instantaneous frequency to describe distortions in waveform (10). Further, we introduce phase-alignment as a solution to comparing full-resolution waveforms between cycles of different durations. Taken together, we establish that the phase-aligned instantaneous frequency profile of an oscillation provides a flexible framework for complete characterisation of oscillatory waveform shape. We demonstrate the utility of this approach by applying it to simulated data and LFP recordings of theta oscillations of behaving mice.

In real data, we observed that theta oscillations have, on average, a fast-ascending and slow descending waveform, in line with previous reports (6, 7, 24, 39). Though this average shape is robust across many cycles, recording sessions and animals; the shape of individual cycles is highly variable. We characterise this variability using PCA to identify a range of shape components, or ‘shape motifs’, which maximally explain the variability in the dataset. The first two PCs quantify the relative durations of the peak and trough (PC-1) and the ascending to descending edge (PC-2). These PCs broadly map onto the features described by the peak-to-trough and ascending-to-descending control point ratios. We show that these theta shape PCs have distinct patterns of covariation with movement speed, theta amplitude and theta cycle duration. Critically, we show that though PC-2 describes less variability overall, it most clearly co-varies with movement speed.

PC-3 and PC-4 capture more complex waveform shapes. We show that the curvature around the extrema of the waveform shape (PC-4) is wider in theta cycles occurring during faster animal movement. This shape is naturally described by instantaneous frequency but not visible to the standard ascending-to-descending and peak-to-trough control-point ratios that we have implemented here. If these shapes were known to be of interest *a priori*, it would be possible to construct specific control point-based measures to identify them. For instance, waveform sharpness can be explored by looking at the differential between the extrema and the samples 5ms before and after (40). However, in real data, we may not know the waveform shape of interest *a priori*, implying that many separate metrics may need to be computed for each cycle. In contrast, the phase-aligned instantaneous frequency can quantify any waveform shape as a within-cycle instantaneous frequency sweep without pre-specifying the features which may be of interest.

The present results demonstrate that single-cycle dynamics in oscillations can be meaningfully estimated using phase-aligned instantaneous frequency, and that specific shape motifs are differentially related to the wider electrophysiological (theta amplitude and duration) and behavioural (movement speed) context. Future models of theta function may consider these dynamics in waveform shape that deviate from a canonical sinusoidal theta template. Given that many sub-processes occur preferentially at different parts of the theta cycle (41), we hypothesise that shape distortion may indicate or reflect a change in the underlying theta-phase-nested sub-processes.

The outlined approach requires that each cycle is smooth in both its waveform and phase profiles, as any jumps or discontinuities will lead to noisy or even negative instantaneous frequency estimates. If the cycle is smooth, we can characterise very large distortions in waveform shape as within-cycle dynamics in instantaneous frequency. Finally, we assume that the features being analysed are well described as oscillations. If the features are non-sinusoidal and non-oscillatory, such as spiking activity, then descriptions using the language of frequency may not be appropriate. With these improvements and caveats in hand, this approach is readily generalisable to other datasets and provides a flexible framework for investigating waveform shape oscillating systems.

In conclusion, the full-cycle waveform of single cycles of hippocampal theta can be quantified and explored with phase-aligned instantaneous frequency. We use this approach to confirm the characteristic fast-ascending waveform of theta oscillations; and to additionally reveal that this is highly variable on the single-cycle level. Moreover, we are able to link this variability with behavioural and electrophysiological states, suggesting that waveform shape is a relevant feature of neuronal oscillations alongside frequency, phase and amplitude. Finally, whilst we have illustrated this approach with hippocampal theta oscillations, it is likely that this methodology will readily generalise to neuronal oscillation in other brain regions, frequency bands and contexts.

## Supplementary Material

Supplementary file

## New & Noteworthy

We propose a novel analysis approach quantifying non-sinusoidal waveform shape. The approach isolates oscillations using the Empirical Mode Decomposition before waveform shape is quantified using phase-aligned instantaneous frequency. This characterises the full shape profile of individual cycles whilst accounting for between cycle differences in duration, amplitude and timing. We validated in simulations before being applied to identify a range of data-driven non-sinusoidal shape motifs in hippocampal theta oscillations.
