# Supplementary material for: Within-cycle instantaneous frequency profiles report oscillatory waveform dynamics
Source: J Neurophysiol. Author manuscript; Available in PMC 2021 Oct 1. (PMC7611760; doi:10.1152/jn.00201.2021)
Supplement: Supplementary file 1 [file EMS133139-supplement-Supplementary_file.pdf]

Within-cycle instantaneous frequency profiles report oscillatory waveform dynamics.

Andrew J. Quinn[1,*], Vítor Lopes-dos-Santos[2], Norden Huang[3,4,5], Wei-Kuang Liang[5,6], Chi-Hung Juan[5,6], Jia-Rong Yeh[4,5], Anna C. Nobre[1,7], David Dupret[2] & Mark W. Woolrich[1]

[1] Oxford Centre for Human Brain Activity, Wellcome Centre for Integrative Neuroimaging, Department of Psychiatry, University of Oxford, OX3 7JX. UK.
[2] Medical Research Council Brain Network Dynamics Unit, Nuffield Department of Clinical Neurosciences, University of Oxford, Oxford, OX1 3TH, UK.
[3] Data Analysis and Application Laboratory, Innovation Centre, The First Institute of Oceanography, Qingdao, China
[4] Pilot National Laboratory for Marine Science and Technology, Qingdao, China.
[5] Cognitive Intelligence and Precision Healthcare Centre, National Central University, Taiwan
[6] Institute of Cognitive Neuroscience, National Central University, Taoyuan City, Taiwan.
[7] Department of Experimental Psychology, University of Oxford, Oxford. OX2 6GG. UK.
[*]correspondence: andrew.quinn@psych.ox.ac.uk

8: Supplemental Methods

Experimental model and subject details

Animals used were male adult (4–7 months old) C57BL/6J mice (Charles River, UK). All animals had free access to water and food in a dedicated housing facility with a 12/12 h light/dark cycle. They shared a cage with their littermates until the surgery. All experiments involving animals were conducted according to the UK Animals (Scientific Procedures) Act 1986 under personal and project licenses issued by the Home Office following ethical review.

Microdrive implantation

Animals were implanted with a 10-12 tetrode microdrive during a surgical procedure performed under deep anesthesia using isoflurane (0.5–2 %) and oxygen (2 l/min), with analgesia (0.1 mg/kg vetergesic) provided before and after. Tetrodes were constructed by twisting together four insulated tungsten wires (12 µm diameter, California Fine Wire) and shortly heating them to bind them together in a single bundle. Each tetrode was attached to a M1.0 screw to enable their independent movement. The drive was implanted under stereotaxic control in reference to bregma (Lopes-dos-Santos et al., 2018). Tetrodes were initially implanted above the CA1 pyramidal layer and their exposed parts were covered with paraffin wax. The drive was then secured to the skull using dental cement. For extra stability, stainless-steel anchor screws had first been inserted into the skull. Two of the anchor screws, which were inserted above the cerebellum, were attached to 50 µm tungsten wires (California Fine Wire) and served as ground and reference electrodes during the recordings. The placement of the tetrodes in dorsal CA1 was confirmed by the electrophysiological profile of the local field potentials in the hippocampal ripple frequency band.

Recording procedures

Recordings commenced following full recovery from the surgery. Each animal was connected to the recording apparatus and familiarized with a high-walled box containing

home cage bedding and with one open-field enclosure (the familiar enclosure) over a period of approximately seven days. During this period, tetrodes were gradually lowered to the stratum oriens of the hippocampal CA1. On the morning of each recording day, tetrodes were further lowered into the pyramidal cell layer in search of multi-unit spiking activity and sharp-wave/ripple events (43). Tetrodes were not moved for at least 1.5 h before recordings started. For each recording day, the animal was exposed to various open-field enclosures including the familiar, which the animal had repeatedly been exposed to before, and a novel enclosure the animal had never seen before. The open-field enclosures differed in shape and in the cue-cards that lined some of the walls. The present study includes a total of six LFP recordings from three mice (including three familiar enclosure and three novel enclosure sessions). At the end of each recording day, tetrodes were raised to the stratum oriens to avoid damaging the pyramidal layer overnight.

Multichannel data acquisition and position tracking

The extracellular signals from the electrodes were buffered on the head of the animal (unity gain op-amps, Axona Ltd) and transmitted over a single strand of litz wire to a dual stage amplifier and band pass filter (gain 1000, pass band 0.1 Hz to 5 kHz; Sensorium Inc., Charlotte, VT), or (in other setups) the electrode signals were amplified, multiplexed, and digitized using a single integrated circuit located on the head of the animal (RHD2164, Intan Technologies, Los Angeles; pass band 0.09 Hz to 7.60 kHz). The amplified and filtered electrophysiological signals were digitized at 20 kHz and saved to disk along with the synchronization signals from the position tracking. LFPs were further down sampled to 1250 Hz for all subsequent analyses. In order to track the location of the animal three LED clusters were attached to the electrode casing and captured at 39 frames per second by an overhead color camera.

8.1: Schematic representation of the mask sift algorithm

```
Algorithm 1: The Mask Sift Algorithm
Result: imf ← mask sift(X)
maskfreq ← [f_1, f_2, f_2, ..., f_N];
maskphase ← [0, π/2, π, 3π/2];
// Loop through mask frequencies
for j = 1 to N do
    // Loop through mask phases
    for k = 1 to 4 do
        // Define mask
        mask ← sin(2 × π× maskfreq [j] × t+ maskphase[k]);
        // Add mask to signal
        tmp ← X + mask;
        // Run sift iterations to convergence
        imf converged ← False;
        while not converged do
            extrema ← find all peaks and troughs in X;
            envelopes ← interpolate envelopes from extrema;
            avg ← take average of upper and lower envelopes;
            tmp ← tmp - avg;
            if avg has converged then
                imf converged ← true;
            end
        end
        // Subtract mask from proto imf
        tmp ← tmp - mask;
        // Store proto imf
        protoimf[k] ← tmp;
    end
    // Next IMF is average across mask phases
    imf[j] ← average protoimf across k;
    // Subtract IMF from input signal
    X ← X - imf[j];
    // Stop sift if X sufficiently close to zero
    if X has converged then
        break
    end
end
```

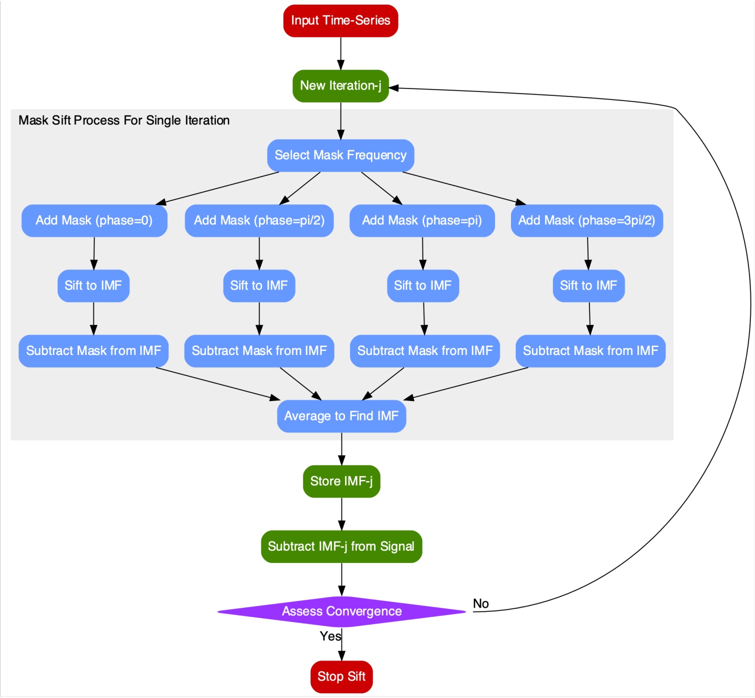

Figure S1: Schematic representation of the mask sift algorithm.
A: Pseudocode for the mask sift
B: Logic flowchart for the mask sift

8.2: Envelope interpolation method

The upper and lower amplitude estimation during the sift is computed using interpolation. A cubic spline interpolator is commonly used for this (15) but it can be prone to overshoot and non-monotonic interpolations in real data (Figure S1). Overshoot (or undershoot) is when the interpolated signal between two points either greatly overestimates the maximum amplitude and non-monotonic interpolation is when the interpolated signal potentially introduces dynamics which are not found in the control points. For example, if three points are strictly ascending then a monotonic interpolation will ensure that the interpolated signal between them is also strictly ascending (33). This extra constraint improves the envelope estimation in real data leading to a cleaner set of IMFs.

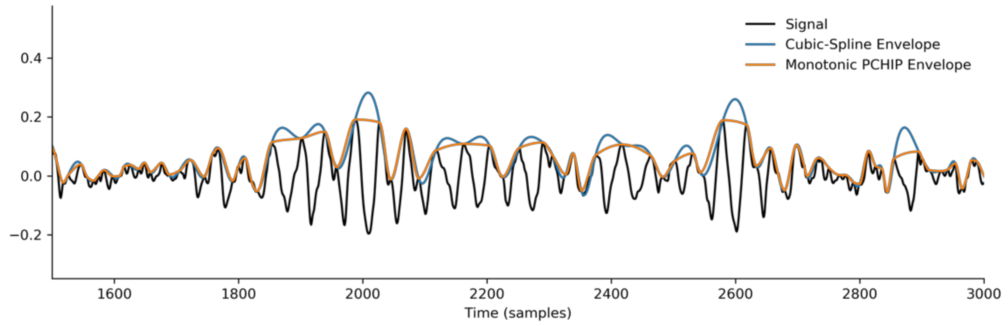

Figure S2 : Illustration of two envelope interpolation methods.

8.3: Mask sift parameter robustness

We ran a supplemental analysis to ensure that our LFP phase-aligned instantaneous frequency results were robust to moderate changes in the masking parameters. 5 minutes of data from a single data recording were repeatedly sifted with jittered masked frequencies. 25 sifts were computed for each of three mask frequency jitter values of 10%, 20% and 30%. For example, in the 10% condition each iteration each mask frequency was randomised to a value drawn from a uniform distribution between 90% and 110% of the original frequency. The results showed that jitter of 10% has a small effect on the phase-aligned instantaneous frequency values, though the centre frequency and shape profile remain consistent across all iterations. Jitters of 20% and 30% have larger effects on both centre frequency and shape on individual iterations, suggesting that some mask frequency combinations are having a large impact on the results. Despite this, the average across all iterations remains strikingly similar. These results indicate that the main waveform results are robust to moderate changes to the masking parameters.

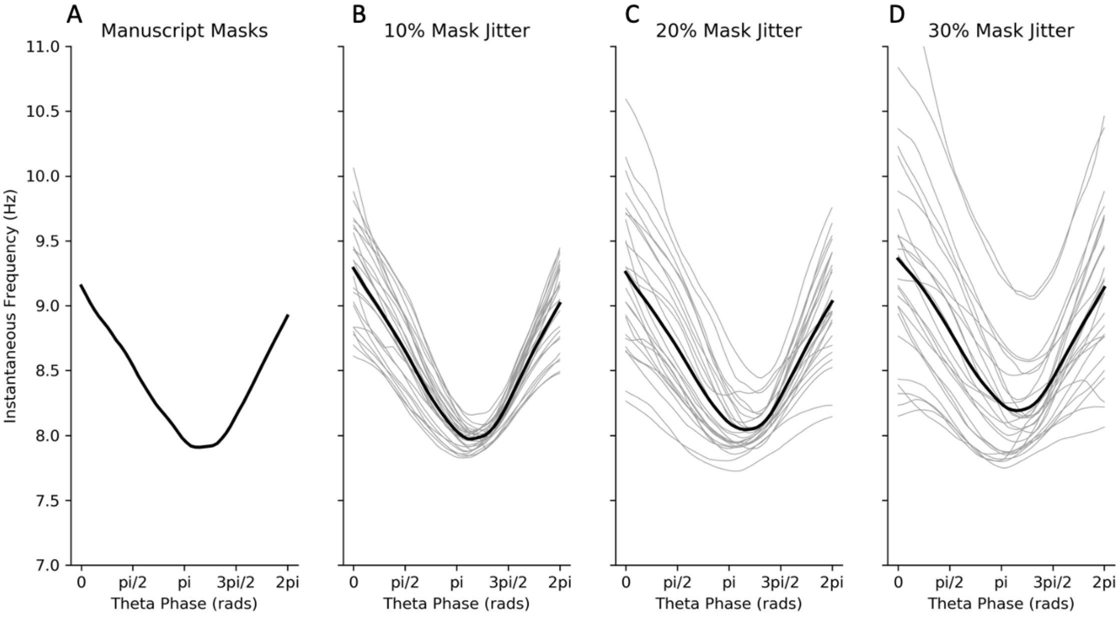

Figure S3 – Phase-aligned instantaneous frequency values across a range of jittered mask frequencies.
A: Instantaneous frequency for mask frequencies used in main analysis
B: Instantaneous frequency for mask frequencies jittered by +/- 10%. Individual iterations are shown in grey and the average in black.
C: As B for jitter of +/- 20%
D: As B for jitter of +/- 30%

8.4: Separation of dynamics at near-harmonic frequency intervals

The theory behind EMD suggests that something is a separate oscillation if it contributes distinct peaks and troughs to the signal. In other words, a component is a harmonic if, when added to another oscillation, it does acts to distort the low frequency signal and does add new extrema to the signal. This can be illustrated by adding together two sinusoids, a base signal at 1Hz and a potential harmonic at 2Hz. When the amplitude of the 2Hz signal is high, the summation contains the same number of extrema as this high frequency component indicating that the EMD will consider the 2Hz signal as distinct dynamics and separate the signal into two IMFs (Figure S3A). When the amplitude of the 2Hz signal is relatively low, the summed signal shows a non-sinusoidal shape and has the same number of extrema as the base signal. The EMD will return a single IMF in this case.

In the LFP data analysis, there is some 20Hz power in the in the HHT shown in figure 6. Though this is close to the frequency of a harmonic component caused by non-sinusoidal shape, in this case it reflects a distinct oscillatory component (similar to the first simulated case). The addition IMF5 to IMF6 introduces additional extrema into the signal – reflecting faster dynamics visible in the raw time course which will be separated into their own IMF.

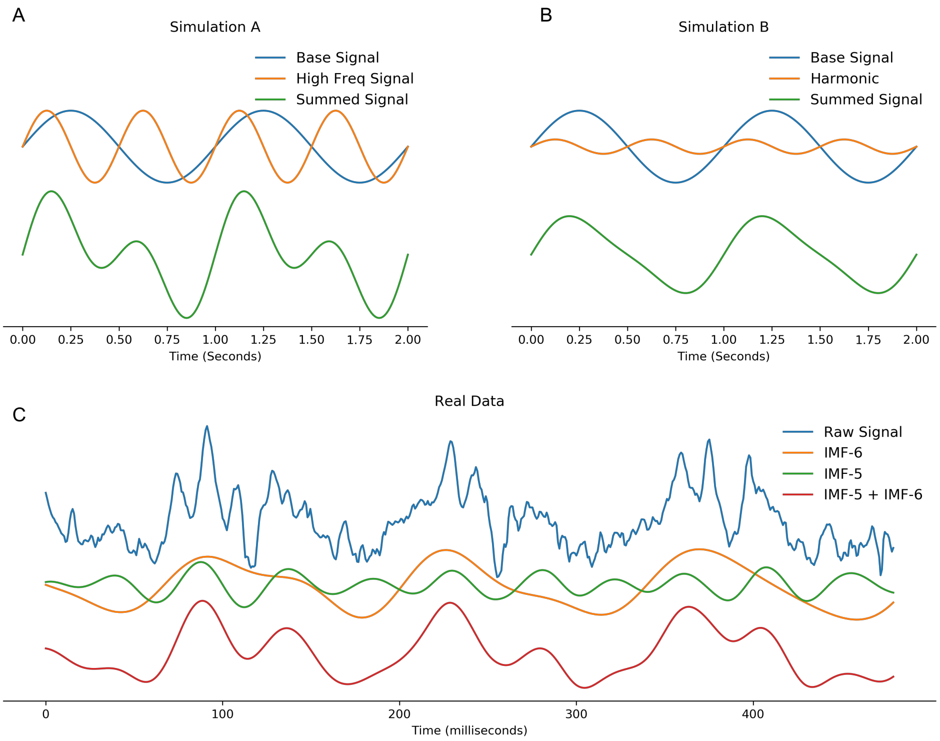

Figure S4 – Distinction between ‘harmonic’ and ‘high frequency’ components in an EMD analysis

A: Simulated example in which a 1Hz base signal is added to a 2Hz high frequency signal. The summed signal has the same number of peaks as the high frequency signal indicating that the 2Hz dynamics constitute a separate oscillation.

A: Simulated example in which a 1Hz base signal is added to a 2Hz harmonic signal. The summed signal has a distorted waveform shape and the same number of extrema as the base signal indicating that the 2Hz signal is a harmonic.

C: A real data segment showing the raw signal, IMF-5, IMF-6 and the sum of IMFs 5 and 6. The sum of the two IMFs contains the same number of extrema as the high frequency component indicating that IMF-5 contains distinct dynamics from IMF-6 in the context of an EMD analysis.

8.5: PCA component selection and reproducibility

The principal components analysis results were validated by computing the split half reproducibility of the PC components across 500 splits. The correlation of component shapes between the separate halves and the proportion of variance explained was computed for each split. The distribution of explained variance for each mode was highly reproducible across the 500 splits and the first four components explained more than 5% of overall variance (Figure S2A). The PC component shapes were also highly reliable for the first four components. The average correlation between components for the two halves of each split was over r=0.95 for the first 5 PCs. Based on these comparisons we carried the first four components forward for further analyses.

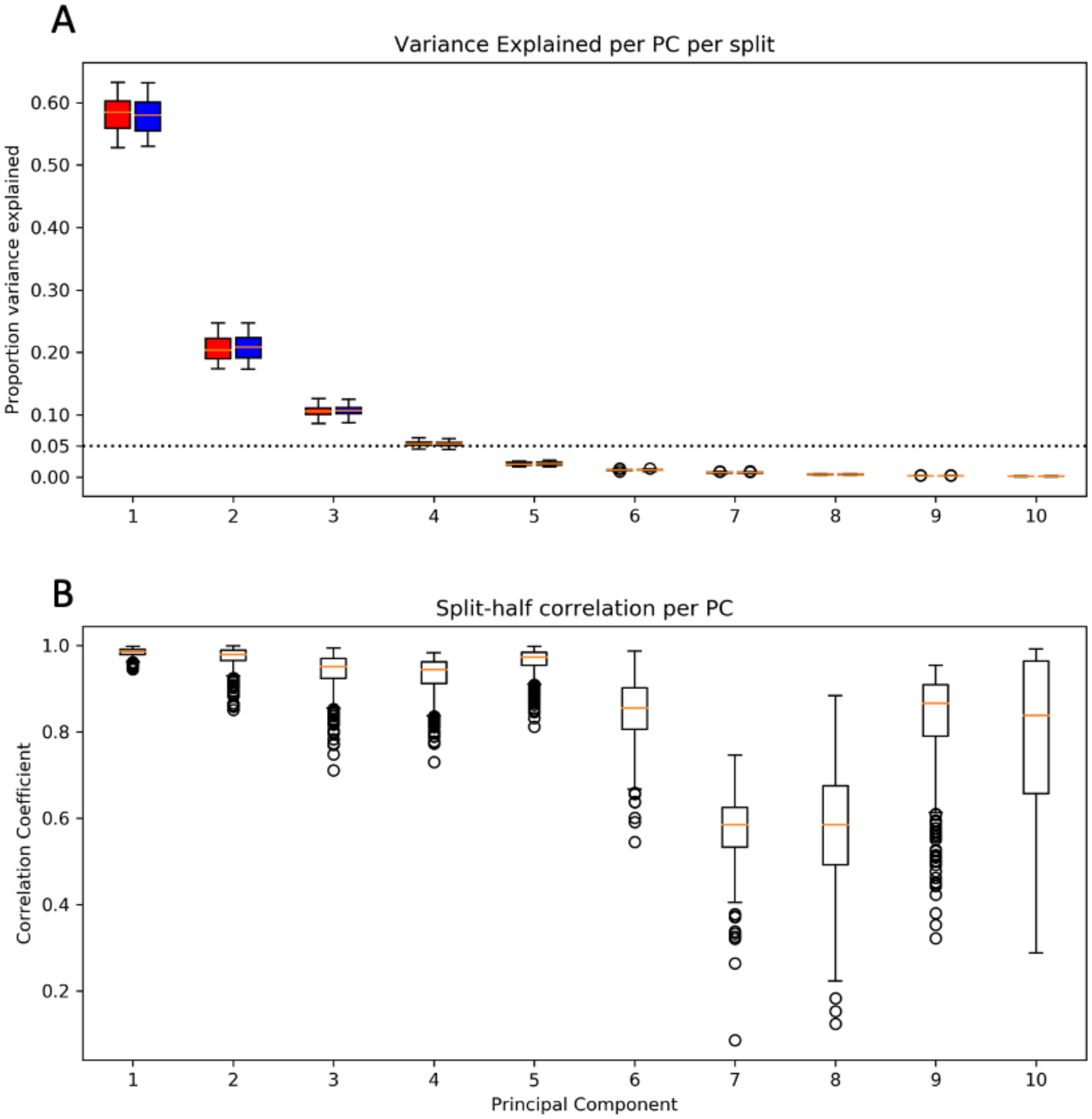

Figure S2 – the variance explained and split-half correlation distributions across 500 split half iterations.
A: the variance explained by each component for each half over the 500 splits. The first half is in red and the second half in blue.
B: The correlation in the component shape between the first and second half of the 500 splits.
